# Volatile Profile of Bee Pollens: Optimization of Sampling Conditions for Aroma Analysis, Identification of Potential Floral Markers, and Establishment of the Flavor Wheel

**DOI:** 10.1002/fsn3.4707

**Published:** 2024-12-25

**Authors:** Mariann Csóka, Rita Végh, László Sipos

**Affiliations:** ^1^ Department of Nutrition Science, Institute of Food Science and Technology Hungarian University of Agriculture and Life Sciences Budapest Hungary; ^2^ Department of Postharvest, Supply Chain, Commerce and Sensory Science, Institute of Food Science and Technology Hungarian University of Agriculture and Life Sciences Budapest Hungary; ^3^ Centre for Economic and Regional Studies, (HUN‐REN KRTK) HUN‐REN Institute of Economics Budapest Hungary

**Keywords:** aroma activity, fingerprinting, headspace–solid phase microextraction, olfactometry, pollen odorants, volatile profile

## Abstract

The volatile profile of bee pollen samples from Central and Eastern Europe was investigated by headspace solid phase microextraction (HS‐SPME) combined with gas chromatography–mass spectrometry‐olfactometry (GC–MS‐O). Sampling conditions were optimized for the extraction of volatiles. Pollen odorants were extracted with six different fiber coatings, five various extraction times, three diverse extraction temperatures and three differing desorption times. The most effective combination was the application of divinylbenzene/carboxen/polydimethylsiloxane (DVB/CAR/PDMS) fiber coating used at 60°C for 30 min for extraction and 1 min for desorption. The optimized method was applied to investigate the volatile profile of 14 pollen samples (three rapeseed, musk thistle, rock‐rose, traveler's joy, dropwort, honey locust, sunflower, red poppy, phacelia, sweet cherry, wild blackberry, and dandelion). The volatile profiles of bee pollens were different and were crucially depended on botanical origin. The aroma activity of the samples was generated by 31.0%–48.3% of total volatiles. The number of the identified odorants were between 75 and 101 in the pollen samples by GC–MS, of which 26–42 were aroma‐active. The volatile organic compounds (VOCs) were classified into 13 different chemical classes. In most pollen, fatty acids were the predominant volatiles (14.87%–50.58%), while in some samples esters were the most abundant odorants (4.09%–45.46%). Panelists confirmed the presence of six main sensory characteristics described as “green/sour”, “fruity”, “spicy/herbal”, “earthy/mushroom”, “sweet/baked/caramel/honey”, and “floral” compounds. These results establish the flavor wheel suitable for the comprehensive sensory description of pollen pellets from individual plant species. All samples contained characteristic odorants that may help in their botanical identification.

## Graphical abstract

The study describes the aroma composition of bee pollens from Central and Eastern Europe. Each pollen possesses its own specific volatile profile, with a number of aroma‐active ingredients. Terpenoids are of great importance for the overall aroma of bee pollen. The results establish the flavor wheel suitable for the comprehensive sensory description of pollen pellets from individual plant species.

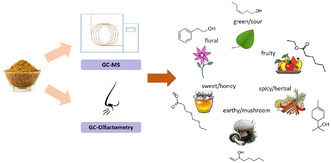

## 1 Introduction

Pollen grains represent the male gametophytes of seed plants, which play a key role in fertilization (Wang and Dobritsa 2018). The process by which pollen grains are transferred to the female reproductive organs of a plant is pollination (Dar et al. 2017). The majority of plant species that contribute to the functioning of ecosystems and the food supply are pollinated by insects (Potts, Imperatriz‐Fonseca, and Breeze 2016). These plants have developed colored petals and a strong scent to attract pollinators (Dar et al. 2017). Pollen‐derived chemical cues originate from pollenkitt, a sticky coating of pollen grains, that contains lipids, pigments, and aroma compounds (Piskorski, Kroder, and Dorn 2011). The latters contribute to attract pollinators, while pollenkitt lipids enable the adhesion of pollen to insect bodies and the formation of pollen pellets by bees (Pacini and Hesse 2005). A wide range of plant species serve as a pollen source for honeybees, encompassing both cultivated and native plants (Radev 2018; Requier et al. 2015). While nectar is mainly the source of carbohydrates, pollen provides proteins and lipids for honeybees (Pietrantuono et al. 2019; Rivest and Forrest 2020). A single plant species constitutes the pollen source for bees at a given time (Barbieri et al. 2020), so each pollen load is monofloral. However, bee pollen obtained by pollen trapping usually consists of several (six on average) plant species, based on the results of 18.000 bee pollen samples collected by 750 beekeepers in Europe (Brodschneider et al. 2021). According to the recommendation of Campos et al. (2008), bee pollen can be referred to as monofloral if the proportion of the predominant pollen is 80% or more. The botanical origin of bee pollen is typically identified through microscopic pollen analysis, although this process requires expertise and is time‐consuming (Marcos et al. 2015). Fingerprinting of bee pollen samples of various origins may be possible by using chemical markers, such as volatile compounds.

Apicultural products, including bee pollen have gained increasing attention of consumers as a food supplement and therapeutic substance in the last decades (Boyacioglu, Tanugur Samanci, and Samanci 2022). The identification and quantification of pollen volatiles represent a key area of research, given the crucial role these compounds play in the sensory characteristics and consumer acceptance of nutraceuticals (Starowicz et al. 2021). Furthermore, pollen volatiles may be used as a fingerprint for botanical origin determination. A comparative analysis of the volatile profiles of pollen and other floral parts, including buds, flowers, petals, and stamens, reveals significant differences in their chemical compositions. Pollen, in particular, exhibits a distinctive volatile profile that is distinct from other floral parts and often displays a lower diversity of volatile organic compounds (VOCs) (Bergström, Dobson, and Groth 1995; Dobson et al. 1987; Dobson, Groth, and Bergström 1996; Dobson and Bergström 2000; Farré‐Armengol et al. 2013; Flamini, Cioni, and Morelli 2003). The scent of the pollen is typically less intense than that of the entire flower, and can only be detected by insects within close range, specifically in the moments preceding and following their landing on the flower (Dobson, Groth, and Bergström 1996; Dobson and Bergström 2000; Farré‐Armengol et al. 2013). The fragrance of pollen appears to originate from the pollenkitt, which is composed mainly of lipids (Dobson 1988; Dobson and Bergström 2000). These lipids can slow the release of volatile components, making the aroma analysis of pollen difficult (Dobson and Bergström 2000). The scent of the pollen (together with other parts of the flower) serves multiple functions. It acts as an attractant for pollinators, conveying information about the availability of food sources. Additionally, it serves a defensive role against predators and pathogens (Dobson, Groth, and Bergström 1996; Dobson and Bergström 2000; Pietrantuono et al. 2019). Bees use the fragrance of pollen to distinguish between flowers, which contain different amounts of pollen (Dobson, Danielson, and van Wesep 1999). Some pollen odorants also have protective or dettering functions: they are probably produced mainly against nonpollinator pollen‐feeding insects and pathogens. The presence of attractive volatiles in the pollen mostly suppresses the deterrent effect of defense components. This phenomenon allows pollination and at the same time protects the pollen against pathogens and herbivores (Dobson and Bergström 2000; Farré‐Armengol et al. 2013). Bees and other pollen‐feeding insects are able to distinguish between different pollen scents and can also differentiate between the aroma of pollen and that of flowers (Dobson 1987; Lunau 1992; Schmidt 1982). The olfactory signal emitted by pollen is a far more species‐specific chemical cue to pollinators than the visual signal provided by flowers (Dobson, Groth, and Bergström 1996).

Based on previous research, pollens contain a large variety of volatile compounds, of which aldehydes, ketons and esters are prevalent (Bertoli et al. 2011; Filannino et al. 2021; Karabagias et al. 2021; Lima Neto et al. 2017; Starowicz et al. 2021). Several studies suggest that the sensory characteristics of bee pollens of different plant species show significant differences (Kostić et al. 2020; Rothschild, Bergström, and Wängberg 2005; Sipos et al. 2020) however, available information on the volatile composition of bee pollens of selected plant species is limited. In Italian *Citrus limon* and *Citrus deliciosa* pollen, terpenes were the prevalent constituents, whereas the exclusive appearance of nonanal may be produced as a protective component against pollen‐feeding animals (Dobson and Bergström 2000; Flamini, Cioni, and Morelli 2003; Flamini, Tebano, and Cioni 2007). In *Cannabis sativa* pollen, 3‐methyl‐butanol and benzyl alcohol were identified as volatile organic compounds (VOCs) exclusive to the pollen, absent from all other plant parts (Rothschild, Bergström, and Wängberg 2005). The dominant volatiles of rapeseed bee pollen were aldehydes with octanal as the most abundant even after drying as well (Bi et al. 2024). This group of volatile lipid oxidation products was also present in lotus pollen with significant intensity and abundance. Subsequent compounds in the profile were ketones and alcohols (Ni et al. 2023). Aldehydes were also predominant in ivy ( *Hedera helix* L.) pollen, with hexanal being prominent (Filannino et al. 2021). This volatile group was also dominant in the aroma of mixed pollen as well (Karabagias et al. 2021; Kaškonienė, Kaškonas, and Maruška 2015; Kaškonienė, Ruočkuvienė, et al. 2015), while in some samples the abundance of esters was outstanding (Keskin and Özkök 2020; Lima Neto et al. 2017).

The determination of the aroma composition of foods is a challenging task due to the chemical diversity of aroma compounds. Aroma analysis consists of four main steps: (1) isolation and concentration, (2) separation, (3) identification and (4) sensory characterization (Starowicz 2021). Headspace‐solid phase microextraction (HS‐SPME) is a commonly used sampling technique for the isolation and concentration of volatiles in foods including bee pollen (Bertoli et al. 2011; Filannino et al. 2021; Kaškonienė, Kaškonas, and Maruška, 2015; Kaškonienė, Ruočkuvienė, et al. 2015; Karabagias et al. 2021; Lima Neto et al. 2017; Ni et al. 2023; Prđun et al. 2021; Starowicz et al. 2021). The SPME method is based on the utilization of a fiber coated with an extraction phase comprising a polymer and/or solid sorbent, which enables the accumulation of the analytes under investigation. The fiber is a part of a special stainless steel needle assembled onto a customized device. In HS‐SPME, the accumulated analytes are recovered by thermal desorption directly into the injector (Bicchi et al. 2012). This technique is straightforward, solvent‐free, rapid, and can be automated. Furthermore, it allows for the use of a range of fiber phases, varying in polarity and molecular size, which facilitates the efficient extraction of analytes (Garvey et al. 2020). Aroma analysis of foods usually involves gas chromatography (GC) that facilitates sensitive, simple and effective separation of volatiles in a range of matrices with complex aromatic profiles (Jadhav 2020). For the detection and identification of volatiles, mass spectrometry (MS) is the most widely applied technique. By using MS, it is possible to screen volatile compounds and compare the obtained mass spectrums with the literature and/or available databases (Starowicz 2021). The evaluation of food aromas can be enhanced through the utilization of olfactometric analysis, which facilitates the utilization of human assessors as a highly sensitive and selective detector of odor‐active compounds. Assessors perceive odor composed of one or more volatile compounds that are present in concentrations above the sensitivity threshold. (Delahunty, Eyres, and Dufour 2006). The application of olfactometric methods for the analysis of aroma‐active constituents in apiculture products is currently limited, only a few research works on the subject are available (Costa, Garruti, and Madruga 2019; Seisonen, Kivima, and Vene 2015; Song and Liu 2018; Tomaszewski 2016; Wardencki et al. 2009; Yang et al. 2010).

The available literature contains only limited information on the volatile profile of monofloral pollens examined in this study. Several aroma studies of mixed (Collin et al. 1995; Karabagias et al. 2021; Kaškonienė, Kaškonas, and Maruška, 2015; Kaškonienė, Ruočkuvienė, et al. 2015; Keskin and Özkök 2020; Lima Neto et al. 2017; Omar et al. 2018; Prđun et al. 2021; Starowicz et al. 2021) and some monofloral (Bergström, Dobson, and Groth 1995; Bi et al. 2024; Dobson 1987; Dobson, Groth, and Bergström 1996; Filannino et al. 2021; Flamini, Cioni, and Morelli 2003; Flamini, Tebano, and Cioni 2007; Ni et al. 2023; Rothschild, Bergström, and Wängberg 2005) pollen are available, but the topic requires further research. To date, no studies have been published on the optimization of headspace‐solid phase microextraction for the analysis of volatile compounds in bee pollen. The first aim of our research is to propose an optimized HS‐SPME‐GC–MS‐O method for identfying pollen volatiles, with particular regard to the aroma‐active constituents. Another objective of our work is to test the method on monofloral bee pollen samples of known plant sources in order to reveal their aroma composition, the odor‐active volatiles and to identify key markers of botanical origin.

## 2 Materials and Methods

### 2.1 Samples and Chemicals

For the optimization of the SPME method, mixed bee pollen was used. This product was purchased from a local supermarket in Budapest and stored in a glass jar in the refrigerator (4°C) until the analysis. Subsequently, the flavor profile of 14 bee pollen samples were determined applying the most effective SPME fiber and the optimal extraction and desorption parameters. For this latter experiment, bee pollens originating from Central and Eastern Europe were obtained from Hungarian beekeepers or purchased in a local retail shop. The products were dried at 38°C ± 2°C, then pollen pellets belonging to different plant species were sorted by color, shape and size. A total of 14 sub‐samples were later subjected to palynological analysis. Following, pollen pellets were grounded and stored at −20° ± 2°C prior to the analysis.

All chemicals used were chromatographic grade. The mixture of n‐alkanes (C_10_‐C_40_) applied for the calculation of retention indexes (RI) were purchased from Sigma‐Aldrich (Steinheim, Germany).

### 2.2 Palynological Analysis

To identify the botanical composition of pollen pellets, microscopic pollen analysis was used. The analysis was conducted by an expert of the Melissopalynological Group of the International Honey Commission (IHC) as follows. Samples were homogenized, then 10 pollen pellets were suspended in 10 mL distilled water and dispersed completely by using a test tube mixer. Thirty microliter of the suspension was transferred onto two slides using a micropipette, and dried on a hot plate. They were covered with glycerin gelatin mixture and glycerin gelatin mixture stained with fuchsine. Pollen grain identification was performed for both slides by examining the entire area of a 20 × 20 mm cover slip. The determination was performed by applying a DELTA Optical binocular light microscope (Delta Optical, Warsaw, Poland) at a 400× magnification.

### 2.3 Optimization of HS‐SPME

Selection of the appropriate SPME fiber is crucial for the complete extraction of pollen volatiles. Different fiber coatings and sampling parameters (extraction time, extraction temperature and desorption time) were investigated to select the most adequate conditions for the flavor analysis of mixed bee pollen. Manual SPME holder was used in these experiments with six different fibers (Supelco, Bellefonte, PA, USA): 100 μm polydimethylsiloxane (PDMS), 85 μm polyacrylate (PA), 75 μm Carboxen‐polydimethylsiloxane (CAR/PDMS), 65 μm polydimethylsiloxane‐divinylbenzene (PDMS/DVB) and 50/30 μm divinylbenzene‐Carboxen‐polydimethylsiloxane (DVB/CAR/PDMS), 10 and 20 mm in length. Prior to the sampling, all fibers were conditioned at 250°C–280°C for 0.5 h depending on the recommendation of the manufacturer. For the extraction of the volatile compounds, 0.5–0.9 g of bee pollen was weighed into 4 mL vials equipped with a screw‐cap syringe valve. The amount of the sample was chosen to leave small room to be saturated, but a gap of sufficient size above the surface for the SPME fibers. The vial containing the pollen sample was placed into a dry heating block of 40°C, 50°C and 60°C to improve the volatility of the analytes, and was allowed to equilibrate for 20 min. Subsequently, the fibers were inserted into the headspace of the sample for 10, 20, 30, 40 and 50 min. Each measurement was repeated three times. After extraction, the fibers were withdrawn and immediately inserted into the GC injection port for desorption at 250°C for 1, 2, and 5 min in the splitless mode. The most efficient SPME fiber was selected based on the number of extracted volatiles and the total peak area.

### 2.4 Instrumental Analysis

#### 2.4.1 GC–MS Analysis

The analysis of the extracted bee pollen volatiles was performed using a Shimadzu GCMS‐QP2010SE gas chromatograph‐mass spectrometer (Shimadzu Corporation, Kyoto, Japan) applying the optimal measuring parameters. The analytes were thermally desorbed from the SPME fiber in the injector port for 1 min at 250°C in splitless mode. The injector was operated with a 6:1 split ratio. The instrument was equipped with a MEGA‐WAX fused silica capillary column (60 m × 0.25 mm × 0.25 μm) (Mega S.r.l., Legnano, Italy). The temperature was programmed from 40°C to 250°C increased at a rate of 5°C min^−1^. The detector was operated in electron impact (EI) ionization mode at 70 eV at 250°C. The detection was performed in the 35–500 mass range at 1666 mass/s scan speed. The temperature of the transfer line was also 250°C. Helium (6.0) was used as the carrier gas with a flow rate of 1.20 mL min^−1^. The results of the measurements were expressed as peak area% assuming all response factors to be unity. It is important to note that the results should not be considered in absolute quantitative terms; however, internal normalization is a standard method in flavor analysis (IOFI 2011). All the analyses were repeated in triplicate and the average of their results is reported.

#### 2.4.2 GC‐O Analysis

For the detection of the odor‐active components, a GCMS‐QP2010SE equipped with a sniffing port (Phaser Pro Olfactory GC Port, GL Sciences B.V., Eindhoven, the Netherlands) was applied. The GC parameters (carrier gas, temperature programme, analysis time) were identical to those employed in the GC–MS analysis. The column effluent was split 3:1 between the olfactory port and mass spectrometer, respectively. To avoid the dehydration of the nasal mucous membranes and prevent olfactory fatigue, the mixing chamber was purged with humidified air at 5 mL/min. Two panelists have evaluated the character and intensity (“Weak”, “Middle”, “Strong”) of the perceived odors. The data were processed by the Olfactory Voicegram software (GL Sciences B.V., Eindhoven, the Netherlands). All measurements were repeated in triplicate.

### 2.5 Identification of Volatile Compounds

The chromatograms and mass spectra were analyzed using the LabSolutions software (Shimadzu Corporation, Kyoto, Japan). The identification of the fragrance constituents was achieved through the utilization of the NIST05 and NIST05s spectrum libraries. The results were confirmed with the calculation of retention indices (RI) for every peaks using n‐alkanes (C10‐C40) (Sigma Aldrich, Steinheim, Germany).

### 2.6 Statistical Analysis

All the measurements were carried out in triplicate and the mean values, standard deviations (SD) and relative standard deviations (%RSD) were calculated. For the quantification of the volatile compounds, internal normalization was applied and the results were expressed as peak area% of the analytes (IOFI 2011). In data tables, the mean values of the parallel measurements were reported. Optimization of the HS‐SPME method parameters (fiber coating, extraction temperature, extraction time, desorption time) was performed in ten replicates, data are expressed as mean and standard deviation. The one‐way ANOVA assessment conditions, the test of normality (Shapiro–Wilk test) and the homogeneity of variance (Levene test) were satisfied. The one‐way analysis of variance (ANOVA) was applied with Tukey‐HSD post hoc test to determine statistical differences between samples (α = 0.05). Letters indicate homogeneous and heterogeneous groups, significant differences are indicated in different letters. The agglomerative hierarchical cluster analysis (Ward's method, Euclidean distances) were carried out for multivariate clustering of aroma groups in pollen samples. Cluster indices (Silhouette index, Calinski and Harabasz index) were used to determine cluster group numbers. The resulting clusters were tested by one‐way ANOVA based on aroma groups (α = 0.05). All data were analyzed using XL‐STAT software, version 2024.1.0 (Lumivero 2024).

## 3 Results and Discussion

### 3.1 Botanical Composition of Pollen Samples

A total of fourteen samples of bee pollen were subjected to analysis in order to determine their botanical origin. The palynological analysis revealed the botanical composition of the pollens. The results of the microscopic examination showed that all samples had a predominant pollen content above 80% (Table 1.), so the pollen samples are considered monofloral according to the ISO bee pollen standard definition (ISO 24382:2023, International Organization for Standardization (IOS) 2023).

**TABLE 1:** The botanical composition of the bee pollen samples.

Sample	Predominant pollen			Minor pollens
	Species	Common name	%	
BN‐1	*Brassica napus*	Rapeseed‐1	96	*Frangula sp., Acer sp., Loranthus europaeus*
BN‐2	*Brassica napus*	Rapeseed‐2	94	*Salix sp*., different fruits
BN‐3	*Brassica napus*	Rapeseed‐3	96	*Acer sp., Loranthus europaeus*
CN	*Carduus nutans*	Musk thistle	94	*Helianthus annuus, Calluna vulgaris, Impatiens sp., Cyanus segetum*
CI	*Cistus incana*	Rock‐rose	97	*Calluna vulgaris, Taraxacum officinale, Thymus sp*.
CV	*Clematis vitalba*	traveler's joy	89	*Plantago sp., Tilia sp., Taraxacum officinale, Trifolium repens*
FV	*Filipendula vulgaris*	Dropwort	95	*Trifolium repens, Helianthus annuus, Chenopodium sp., Carduus nutans, Papaver rhoeas, Impatiens sp., Leucanthemum vulgare*
GT	*Gleditsia triacanthos*	Honey locust	99	*Tilia sp*.
HA	*Helianthus annuus*	Sunflower	97	*Taraxacum officinale*
PR	*Papaver rhoeas*	Red poppy	94	*Phacelia tanacetifolia, Ligustrum sp., Tilia sp., Convolvulus arvensis*
PT	*Phacelia tanacetifolia*	Phacelia	100	—
PA	*Prunus avium*	Sweet cherry	96	*Salix sp*.
RF	*Rubus fruticosus*	Wild blackberry	98	Not identified
TO	*Taraxacum officinale*	Dandelion	84	*Salix sp., Brassica napus*, different fruits

### 3.2 Optimization of the HS‐SPME Method

#### 3.2.1 Selection of Fiber Coating

**FIGURE 1:**
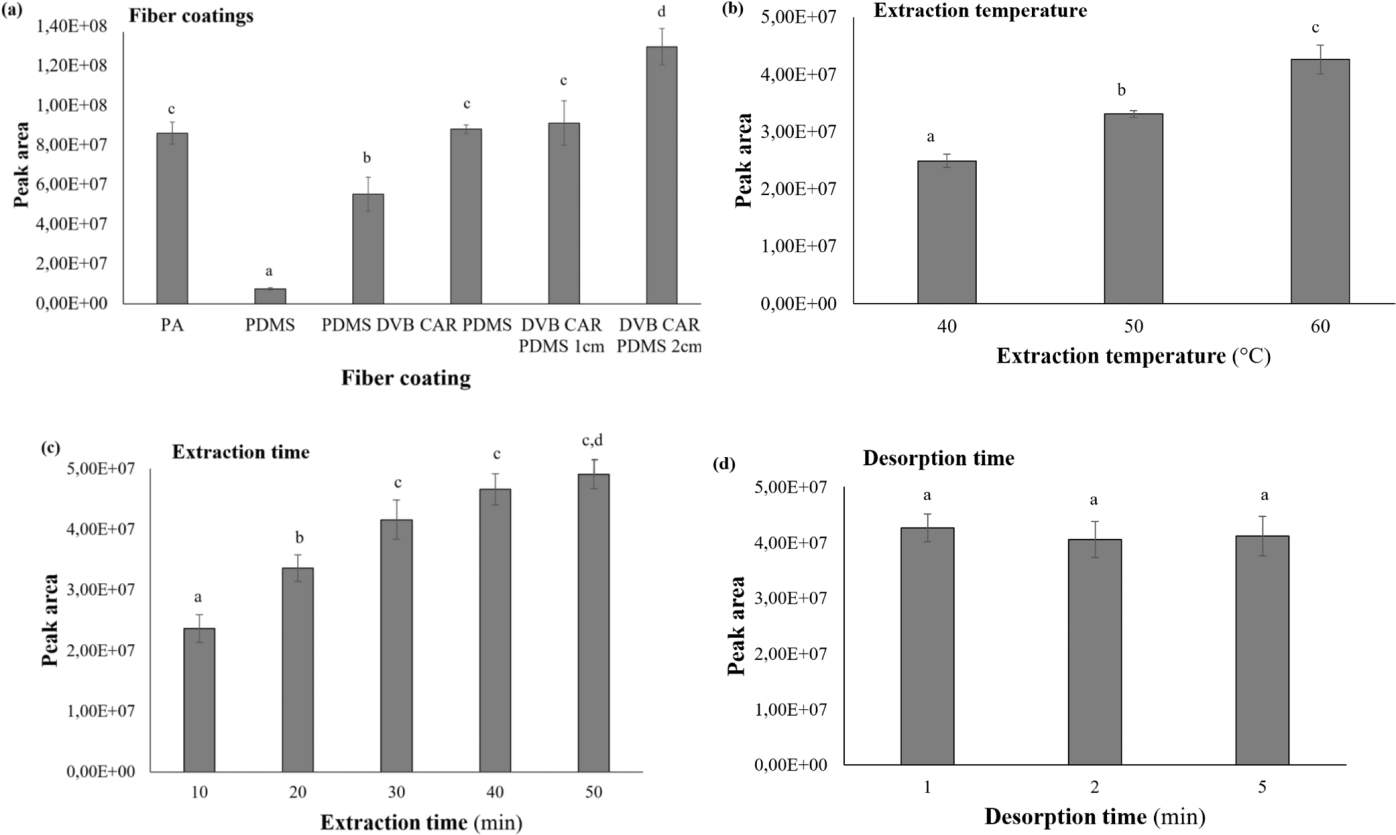
SPME optimization on bee pollen volatiles. (a) Fiber coatings (b) Extraction temperature (°C) (c) Extraction time (min) (d) Desorption time (min); *Letters indicate homogeneous and heterogeneous groups, significant differences (p < 0.05) are indicated in different letters*.

The type of fiber coating is a crucial determinant of the analytical performance of SPME, as it influences the selectivity of extraction (Al‐Taher and Nemzer 2020). In addition, the experiment examined fibers of three distinct coating types: polar, non‐polar and mixed‐phase. The results were evaluated in terms of total peak areas and the number of extracted volatiles. The six applied fiber coatings showed different efficiency to extract volatiles from bee pollen sample. The highest quantity of extracted odorants, as expressed in total peak area, was observed in the case of fiber coating DVB/CAR/PDMS 20 mm in length, while the lowest for PDMS (Figure 1a). These fibers extracted 68 and 55 volatiles, respectively.

Extraction with the nonpolar PDMS fiber was very weak compared to the other five fibers (Figure 1a). The results are readily attributable to the different characteristics of the fiber types. PDMS is the most common non‐polar coating material, which is applied for the analysis of volatiles and non‐polar semivolatiles (Vas and Vékey 2004). The most intense compound classes were acids (30.96%) and hydrocarbons (20.11%), with heptanoic acid representing the most abundant (13.46%) constituent. The nonpolar PDMS coating demonstrated notable efficacy in the absorption of terpenes (16.72%). Additionally, the sulfur‐containing volatile ratio was the highest among the examined fibers, at 7.15%.

Polyacrylate is a moderately polar coating with high affinity for polar semivolatiles, aromatic compounds and oxygenated analytes (Shirey 2012). Alcohols (38.19%), nitrogen‐containing constituents (32.82%), hydrocarbons (10.23%) and acids (9.53%) were the most abundant volatile groups applying polyacrylate fiber for extraction. The chromatogram was dominated by 1,3‐butanediol (37.92%). This polar coating was especially sensitive for 4‐octadecylmorpholine (32.59%), which compound was not considered a characteristic bee pollen volatile, since it was identified formerly in tomato (Figueira et al. 2014), in the fruit of *Geonoma pauciflora* (Araujo, Do Nascimento, and Mendes 2011) and was shown to be produced by *Bacillus subtilis* G8, possessing antifungal properties that will be crucial to its survival (Liu et al. 2008). Due to its polar character, this fiber was not susceptible to terpene volatiles. Only geranyl acetone, an isoprenoid compound, was absorbed (0.16%). The polyacrylate coating exhibited the lowest sensitivity among the fibers tested in terms of the number of extracted odorants. Both PDMS and PA phases are absorbent‐type fiber coatings, the odorants dissolve and diffuse into the coating material (Vas and Vékey 2004). Although the total peak area of volatiles absorbed by the PDMS fiber was considerably lower than that of the PA coating, the former extracted a greater number of odorants (55 vs. 44). Mixed fiber coatings like PDMS/DVB, CAR/PDMS and DVB/CAR/PDMS extract volatiles by physical capture, they migrate into the pores of the adsorbent when extracted. In case of adsorption, the displacement of certain volatiles may occur as a consequence of the restricted availability of free spaces on the surface of the fiber coating. This process exerts an influence on the percentage of adsorbed volatiles (Majcher and Jeleń 2009).

PDMS/DVB is used primarily for the extraction of semivolatiles and analytes with higher molecular weight (Shirey 2012). The number of the extracted volatiles was 101, which represents the highest value among the examined fiber coatings. The volatile fraction adsorbed by this fiber coating was predominantly composed of acids, with acetic acid exhibiting the most intense peak (11.54%). The coating was quite sensitive for oxygen‐containing heterocycles (16.56%), mostly for lactones. Among benzenoid volatiles (11.62%), benzaldehyde was the most abundant (9.89%). The volatile fraction was also composed of significant quantities of ketones (10.65%) and hydrocarbons (9.23%), particularly those with higher molecular weight.

CAR/PDMS fiber is a good choice for extracting small molecules, because the pore size of Carboxen does not allow greater molecules to enter the micropores (Pillonel, Bosset, and Tabacchi 2002). The application of the bipolar coating resulted in the dominance of acid components (80.08%) in the aroma profile, predominantly those with a low carbon number; the peak area of acetic acid alone reached 67.23%. The proportion of other chemical classes was relatively low: acids were followed by oxygen‐containing heterocycles (7.20%), ketons (3.78%) and compounds with benzene ring (3.25%). This fiber showed slight sensitivity to terpenoid volatiles, with five compounds identified at a peak ratio of 1.15%. Among the multilayer fibers, Carboxen‐PDMS demonstrated the lowest capacity for the adsorption of fragrance compounds, with a total of 53 volatiles adsorbed.

The dual‐coated DVB/CAR/PDMS fiber is composed of a layer of PDMS/DVB over a layer of CAR‐PDMS so it combines the favorable properties of these fibers: it can extract analytes with low and higher molecular weights as well (Al‐Taher and Nemzer 2020). It is recommended for flavor and odor extraction for volatiles and semi‐volatiles (Ducki et al. 2008). The 10 and 20 mm DVB/CAR/PDMS fibers exhibited notable sensitivity to acids (78.41% and 73.62%). This sensitivity was particularly pronounced in the presence of acetic acid. A large number of terpenes and heterocyclic compounds were adsorbed by the dual‐coated fibers. The coatings demonstrated a high affinity for these odorous, frequently character‐defining volatile constituents. The number of the extracted volatiles was found to be 65 and 68, respectively, for the shorter and longer coatings. The DVB/CAR/PDMS fiber, 20 mm in length, was found to be the most efficient of the fibers tested, with its outstanding adsorbed volatiles expressed in total peak area (Figure 1a). This result was in agreement with a number of other studies where this fiber has also been shown to be the most effective for extracting food volatiles from various matrices (Al‐Taher and Nemzer 2020; Arcari et al. 2017; Bogusz Junior et al. 2011; Ducki et al. 2008; Ferreira, Perestrelo, and Câmara 2009; Fiorini et al. 2014; Geng et al. 2019; Jouquand et al. 2008; Lorenzo 2014; Majcher and Jeleń 2009; Meng et al. 2019; Murat et al. 2012; Nogueira et al. 2018; Plutowska et al. 2011; Raza et al. 2019; Wang et al. 2017, 2019; Zhang et al. 2021). A comparative analysis of the DVB/CAR/PDMS coatings of varying lengths demonstrated that the longer fiber exhibited a higher total peak area and a greater number of adsorbed volatiles. Therefore, the aforementioned coating was selected for further investigation of the volatile compounds present in bee pollen.

#### 3.2.2 Optimization of Extraction Conditions

In addition to the fiber coating, the extraction conditions represent another crucial parameter in terms of the efficiency of the sampling process. Three temperatures (40°C, 50°C and 60°C), five extraction times (10, 20, 30, 40 and 50 min) and three desorption times (1, 2, and 5 min) were examined to select the most effective conditions for the extraction of bee pollen volatiles.

The extraction of volatile compounds is significantly influenced by temperature. At elevated temperatures, the thermal movement of molecules is enhanced, facilitating their release from the matrix (Meng et al. 2019). This phenomenon was also observed in this experiment: the increase of temperature from 40°C to 60°C improved extraction efficiency; the application of 60°C has resulted in the largest total peak area (Figure 1b). The number of volatile compounds detected increased with the elevation of the extraction temperature, reaching 35, 45, and 59, respectively. In addition to the number of volatile components adsorbed, the peak areas of numerous odorants are also increased at elevated extraction temperatures. For example, acids and lactones are much better extracted at 60°C, compared to the lower temperatures. For the further experiments, extraction temperature of 60°C was selected. The application of higher temperature for extraction is not recommended, because of the chemical changes occurring in the sample. Heat‐sensitive volatiles may undergo degradation or other undesirable transformations and reactions (Ducki et al. 2008; Meng et al. 2019; Cremer and Eichner 2000).

The effect of the extraction time was also examined in this study. The efficiency was quantified in terms of the number of the extracted volatiles and the total peak area. As the extraction time increased, the total peak area also grew (Figure 1c). However, the number of adsorbed volatiles remained relatively consistent, with 45, 49, 60, 58, and 55 volatiles detected with each successive extraction time. As with the effect of extraction temperature, an extension of the extraction time also resulted in a significant increase in the peak area of acids with larger molecular weight and lower volatility. An extended extraction period at a higher temperature may prove an effective approach; however, it may also result in the formation of thermal degradation products. Furthermore, a brief sampling period is essential for the accurate interpretation of the data. Therefore, it is essential to achieve an equilibrium between the efficiency of the extraction process and the minimization of the extraction time. As a result of the experiments, extraction at 60°C for 30 min was selected for further measurements.

Furthermore, desorption time may also exert an influence on HS‐SPME sampling. The adsorbed volatiles were desorbed in the GC injector port for 1, 2, and 5 min at 250°C in the splitless mode. The results demonstrated that desorption time had no significant impact on the amount of adsorbed volatiles (Figure 1d), so the shortest time period (1 min) was selected. It is recommended that the application of a lengthy desorption time at a high temperature be avoided, as this may result in a reduction in the lifespan of the fiber coating.

In conclusion, the optimal conditions for the extraction of bee pollen volatiles were found to be the utilization of a 50/30 μm DVB/CAR/PDMS fiber coating with a length of 2 cm, extraction at 60°C for 30 min and a desorption time of 1 min.

### 3.3 Volatile Composition of the Pollen Samples

#### 3.3.1 Volatile Profile of Pollen Samples

Regarding the volatile composition of apiculture products, the majority of available data pertain to honey. Consequently, this research could prove a valuable addition to the existing body of knowledge regarding the aroma compounds present in pollen. In this experiment, the volatile profile of fourteen bee pollen samples was investigated, employing the optimized sampling parameters. The fragrance constituents in the bee pollen samples were extracted by HS‐SPME applying DVB/CAR/PDMS fiber and analyzed by GC–MS‐O. A total of 296 aroma components were identified in the 14 samples. Applying the same DVB/CAR/PDMS coating, Starowicz et al. (2021) identified 33 volatile compounds in bee pollen sample from Poland, Karabagias et al. (2021) found 25 odorants in Greek pollen, Filannino et al. (2021) detected 106 compounds in Italian ivy ( *Hedera helix* L.) pollens and Prđun et al. (2021) extracted 54–62 fragrance constituents from Croatian unifloral pollen samples. Using PDMS fiber, Flamini, Cioni, and Morelli (2003) absorbed 22 odor components from *Citrus deliciosa* pollen, while Lima Neto et al. (2017) identified 138 volatiles in stingless bee pollen. Kaškonienė, Kaškonas, and Maruška, 2015 detected a large number (103) of volatile compounds in Lithuanian mixed pollen using PDMS/DVB fiber. As shown in Table 2, volatile constituents of the pollen samples were classified into 13 different chemical classes: terpenoids, sulfur‐, and nitrogen‐containing compounds, O‐heterocycles, benzenoid volatiles, aldehydes, ketones, alcohols, esters, acids, lactones, hydrocarbons and others.

**TABLE 2:** Volatile headspace compounds (peak area%) identified in bee pollen samples.

RI	Compound	Samples^a^													
		BN‐1	BN‐2	BN‐3	CN	CI	CV	FV	GT	HA	PR	PT	PA	RF	TO
**Terpenes**		**7.61**	**3.01**	**4.54**	**11.62**	**3.58**	**4.97**	**9.98**	**5.36**	**33.14**	**19.06**	**4.54**	**17.59**	**6.02**	**9.94**
1007	*α*‐Pinene	0.24	0.06	0.13	0.73	0.11	0.17	0.43	0.12	19.60	0.26		0.30	0.29	0.42
1043	Camphene									0.20					0.12
1080	*β*‐Pinene	0.08		0.07	0.21	0.04	0.07	0.12		0.45	0.16		0.11	0.07	0.31
1093	Sabinene	0.19		0.08	0.34	0.04	0.11	0.24	0.11	2.85	0.22	0.07	0.27	0.12	0.41
1135	*β*‐Myrcene	0.24			0.36	0.09	0.12	0.50		0.20	0.27		0.41	0.19	0.28
1139	Linalyl anthranilate					0.05				0.09					
1143	*α*‐Limonene diepoxide						0.14							0.43	
1142	*α*‐Phellandrene	0.08			0.08			0.18			0.19		0.13		0.14
1154	*α*‐Terpinene									0.52					
1178	D‐Limonene	4.53	1.52	2.54	6.50	1.97	2.37	6.39	1.93	2.50	3.22	1.67	5.43	3.07	3.72
1188	*β*‐Phellandrene									0.12					
1194	Eucalyptol			0.18											0.92
1228	*γ*‐Terpinene				0.42	0.18		0.29		1.15	0.15		0.32	0.21	
1232	Ocimene	0.31					0.20				0.14				
1256	*β*‐Cymene									0.96					
1267	*α*‐Terpinolene									0.15					
1295	4,8‐Dimethyl‐3,7‐nonadien‐2‐ol									0.02					
1360	Farnesane									0.07					
1363	Phytane	0.75			0.80	0.21	0.41	0.58			0.38		0.55	0.35	0.64
1437	Tetrahydrolinalool									0.03					
1465	*cis*‐Linalool Oxide		0.20							0.43		1.49			
1471	Dihydromyrcenol												0.02		
1513	*α*‐Copaene								2.14						
1553	Isothujol											0.12			
1555	Linalol	0.11									0.19				0.15
1564	Lilac aldehyde A												1.00		
1587	Lilac aldehyde B												1.07		
1605	Borneol, acetate									0.43					0.33
1618	Lilac aldehyde C												0.05		
1619	*δ*‐Cadinol									1.01					
1621	Hotrienol	0.49			0.54	0.07		0.15	0.17			0.05			
1624	*δ*‐Cadinol									0.04					
1625	Caryophyllene			0.06	0.44	0.04					0.15				
1630	4‐Terpineol									0.16					
1655	*β*‐Cyclocitral												0.93		
1658	1‐Menthol									0.11					
1680	Ethyl iso‐allocholate										1.04				
1701	*β*‐Chamigrene									0.76					
1703	*γ*‐Gurjunene				0.11				0.15						
1712	*β*‐Citral										0.27				
1722	l‐*α*‐Terpineol			0.18											0.22
1719	*trans*‐2‐Pinanol										0.23				
1721	Isoborneol			0.11											
1728	Borneol														0.29
1740	Geranyl acetate										0.27				
1752	Lilac alcohol A												1.69		
1756	Epoxylinalol		0.13	0.09	0.19							0.52			
1756	Perilla aldehyde									0.32					
1761	Citral										0.29				
1761	Verbenone						0.12								
1764	*cis*‐Verbenol									0.16					
1773	Lilac alcohol B												1.08		
1775	Dihydrocarveol									0.19					
1778	D‐Carvone	0.23			0.48	0.24	0.46	0.62			0.90			0.40	0.50
1779	*γ*‐Muurolene								0.39						
1794	*α*‐Curcumene	0.13			0.14	0.05	0.09	0.14			0.18		0.16	0.07	
1802	Artemisia ketone		0.29	0.69					0.18			0.38			
1809	Lilac alcohol C												0.52		
1812	*cis*‐Geraniol		0.19								10.19				
1849	Lilac alcohol D												0.53		
1874	Geranyl acetone	0.23	0.49	0.29	0.27	0.49	0.36	0.28	0.16	0.62	0.36	0.07	0.32	0.47	0.46
1883	*α*‐Ionone		0.13	0.12			0.11						0.71	0.18	0.38
1969	*β*‐Ionene						0.09	0.07					1.17		0.23
2012	Caryophyllene oxide				0.01										
2022	*β*‐Ionone epoxide						0.17						0.73	0.17	
2041	Nerolidol														0.43
2066	Epoxy‐linalooloxide											0.18			
2128	Hexahydrofarnesyl acetone						0.08						0.09		
**Sulfur‐containing compounds**		**0.53**	**1.00**	**0.64**	**1.25**	**0.28**	**0.25**	**1.09**	**0.80**	**0.09**	**0.19**	**0.07**	**4.07**	**0.20**	**0.62**
878	Dimethyl sulfide	0.33	1.00	0.64	0.19	0.09		0.65	0.09	0.09	0.07	0.07	2.85	0.20	0.09
1057	Dimethyl disulfide	0.12													
1383	Dipropyl disulfide	0.08			0.12			0.14			0.12		0.16		0.03
1493	1.1‐Dimethyl‐decyl‐mercaptan				0.93	0.19									
1496	2‐Methyl‐2‐undecanethiol							0.31							0.50
1641	Dimethyl Sulfoxide						0.25		0.71				1.06		
**Nitrogen‐containing compounds**		**1.32**	**0.58**	**0.20**	**1.01**	**0.29**	**0.44**	**0.76**	**0.84**	**0.39**	**1.27**	**0.24**	**1.18**	**1.20**	**0.73**
848	1,2‐Propanediamine	0.48	0.41	0.20	0.59	0.13	0.20	0.42	0.36	0.39	0.61		0.46	0.45	0.29
1001	Methyl isocyanide	0.09				0.06	0.09	0.14	0.05						
1521	Ammonium oxalate										0.24				
1787	3‐Methyl‐1H‐pyrazole											0.24			
1914	1‐Acetylpyrrolidine														0.28
1955	Benzyl nitrile		0.17												
1994	2‐Acetylpyrrole	0.39			0.42	0.10	0.15	0.19	0.42		0.42		0.72	0.75	0.12
2088	2‐Pyrrolidinone	0.36													0.04
**Oxygen‐containing heterocyclic compounds**		**2.76**	**2.17**	**1.62**	**8.47**	**0.76**	**0.98**	**3.32**	**5.62**	**5.73**	**3.89**	**2.30**	**3.06**	**3.03**	**1.07**
913	2‐Methylfuran											0.06			
955	2‐Ethylfuran											1.90			
1209	2‐Pentylfuran		0.17	0.13	0.28	0.11		0.24	0.61	0.28	0.23		0.44	0.16	0.23
1254	7‐Azabicyclo[4.2.2]deca‐2,4,9‐trien‐8‐one		0.31												
1307	Furaneol	0.13		0.14											
1347	Tetramethyloxirane											0.34			
1485	Furfural	0.65	1.43	1.09	4.80	0.20	0.42	2.32	3.44	5.25	1.48		0.67	1.16	0.57
1534	2‐Acetylfuran			0.11	0.15		0.06	0.07	0.36				0.08		
1609	5‐Methylfurfural				0.50				1.05						
1676	2‐Furanmethanol	0.61			0.58						0.48			0.35	
1807	5‐Ethyl‐2(5H)‐furanone	0.61												0.07	
1891	Furaneol														
2264	2,3‐Dihydro‐3,5‐dihydroxy‐6‐methyl‐4H‐pyran‐4‐one	0.76	0.21	0.16	2.16	0.45	0.51	0.69	0.16	0.19	1.69		1.65	1.30	0.27
**Benzene ring compounds**		**5.26**	**3.73**	**3.27**	**7.44**	**2.91**	**3.09**	**5.44**	**2.12**	**3.06**	**6.20**	**1.82**	**6.66**	**3.33**	**6.37**
1026	Toluene	1.08	0.29	0.17	1.01	0.29	0.24	0.83	0.17	0.40	0.42	0.07	0.73	0.31	0.85
1034	(2‐Methyloctyl)benzene			0.06							0.11			0.04	
1104	Ethylbenzene	0.61	0.15	0.10	0.74	0.16	0.24	0.57	0.14	0.31	0.44	0.15	0.65	0.26	0.47
1116	*o*‐Xylene	0.08	0.23	0.21	0.76	0.21	0.18	0.56							
1118	*p*‐Xylene	0.24	0.76	0.50	1.26	0.38	0.40	1.07	0.16	0.30	0.31		0.58	0.26	0.57
1123	*m*‐Xylene	1.26			0.16	0.04	0.07	0.23	0.58	0.91	1.11		1.65	0.70	1.37
1251	Styrene	0.75	0.30	0.34	0.83	0.38	0.26	0.76	0.23	0.35	0.38	0.20	0.77	0.30	0.67
1258	2‐Ethyl‐1,4‐dimethylbenzene	0.45		0.28	0.73	0.62	0.24	0.69	0.33		0.46	0.15	0.61	0.35	0.62
1434	1,3‐Dichlorobenzene		0.29	0.24	0.31	0.10	0.13	0.37			0.28		0.36	0.23	0.18
1561	Benzaldehyde	0.45	0.80	0.67	1.37	0.42	1.07		0.50	0.37	2.02	1.10	1.23	0.73	1.08
1678	Benzyl oleate														0.35
1693	Acetophenone	0.03			0.02	0.09					0.09		0.08		
1784	Linalyl phenylacetate			0.11											
1896	Benzyl Alcohol	0.15	0.23	0.12	0.25	0.09	0.10			0.13	0.19			0.09	0.21
1931	Phenylethyl Alcohol	0.17	0.38	0.17			0.06			0.28		0.15		0.07	
1934	(2‐Ethyloctyl)benzene							0.37							
1968	p‐Methylguaiacol										0.03				
2016	Methyleugenol					0.15									
2071	*p*‐Anisaldehyde						0.10								
2082	Cinnamylaldehyde		0.30	0.24							0.36				
2163	Eugenol			0.05											
**Alcohols**		**7.62**	**9.23**	**9.06**	**15.10**	**6.21**	**7.52**	**14.38**	**9.69**	**11.34**	**13.58**	**7.32**	**14.55**	**11.55**	**9.64**
942	4‐Methoxy‐1‐butanol							0.48							
946	Ethyl alcohol	6.14	3.80	5.07	12.03	4.86	6.63	11.03	4.40	5.79	10.44	2.06	11.06	7.37	8.36
1047	2‐Methylpentanol		0.08												
1130	1‐Penten‐3‐ol	0.21	1.10	0.33					0.68	0.10	0.06	2.63			
1228	1‐Pentanol		0.39	0.39					0.28			0.34			0.25
1290	3,4‐Dimethyl‐2‐hexanol				0.46										0.42
1291	4,8‐Dimethyl‐3,7‐nonadien‐2‐ol									0.64					
1307	*(Z)‐*2‐Penten‐1‐ol		0.15									1.10			0.08
1341	1‐Hexanol		0.09	0.09		0.02					0.29		0.08		
1363	2‐Heptanol											0.06			
1378	*(Z)‐*3‐Hexen‐1‐ol		0.16	0.19											
1405	*(E)‐*2‐Nonen‐1‐ol	0.22			0.58	0.45		1.06	0.23	0.40	0.91	0.49	0.68	0.55	0.35
1432	3,7‐Dimethyl‐3‐octanol									0.42					
1460	6‐Methyl‐5‐hepten‐2‐ol		0.54												
1472	2,6‐Dimethyl‐7‐octen‐2‐ol			0.10		0.03		0.22	0.16		0.09		0.16	0.14	
1492	2‐Ethylhexanol	0.74								1.39					
1521	2‐Decanol	0.32									0.14			0.54	
1543	1‐(2‐Methoxypropoxy)‐2‐propanol														
1540	2‐Methyl‐2‐hexanol										0.82				
1552	3‐Methyl‐2‐hexanol		0.40	0.22				0.21		0.58					
1553	2,3‐Butanediol				0.62				1.01						
1564	1‐Octanol		0.45	0.29		0.38		0.48	0.39	0.61	0.56				
1589	[R‐(R*,R*)]‐2,3‐Butanediol		1.09	0.56	1.41	0.35	0.57	0.90	2.54	0.98				0.82	
1620	2,6‐dimethyl‐3,7‐Octadiene‐2,6‐diol		0.39	0.60							0.26	0.16	0.49	0.13	0.17
1626	2‐Octen‐1‐ol											0.05			
1634	2,6‐Dimethylcyclohexanol												1.57		
1670	1‐Nonanol									0.26					
1677	2‐Ethyl‐1‐hexanol									0.03					
1686	Dipropylene glycol		0.36												
1687	1‐[2‐(Allyloxy)‐1‐methylethoxy]‐2‐propanol			0.79											
1696	Tripropylene glycol		0.24	0.37											
1760	*(E,Z)‐*3,6‐Nonadien‐1‐ol			0.06											
1770	2‐Methylhexadecanol									0.13					
1812	2‐Ethyl‐2‐hexen‐1‐ol											0.43			
1897	2,5‐Dimethyl‐3‐hexyne‐2,5‐diol												0.23		
1970	3‐Isopropyl‐4‐methyl‐1‐pentyn‐3‐ol					0.12									
**Aldehydes**		**3.23**	**6.01**	**5.01**	**1.15**	**1.42**	**0.83**	**4.17**	**16.83**	**3.00**	**2.99**	**25.30**	**3.52**	**1.71**	**1.48**
868	Acetaldehyde		0.22	0.13							0.09	0.29	0.31	0.16	
887	Propanal											2.74			
891	2,4‐Dimethylpentanal	0.13													
919	Butanal											0.30			
939	3‐Methylbutanal	0.34	1.14	2.14							0.80	0.24			
980	Pentanal	0.14							0.24			1.50			0.09
1023	3‐Methylpentanal														0.15
1040	2‐Butenal						0.03					0.42			
1062	Hexanal	0.73	1.32	0.95	0.83	0.20	0.23	0.78	2.99	0.84	0.69	10.80	1.14	0.69	0.72
1146	2‐Methyl‐2‐pentenal		0.07									0.21			
1210	*(E)‐*2‐Hexenal											3.15			
1284	Octanal	0.38	0.42	0.47		0.13	0.16	0.41	1.72	0.44	0.55	0.38	0.44	0.25	0.26
1331	*(Z)‐*2‐Heptenal											0.19			
1402	Nonanal	0.89	2.36	0.96	0.31	1.09	0.41	2.99	11.83	0.86	0.87	0.47	0.58	0.60	0.26
1431	*(E,E)‐*2,4‐Hexadienal											0.53			
1494	2‐Undecenal												0.90		
1515	Decanal	0.60	0.48	0.36						0.86		0.19	0.16		
1521	*(E,E)‐*2,4‐Heptadienal											3.34			
1609	*(E,Z)‐*2,6‐Nonadienal											0.09			
1695	2‐Butyl‐2‐octenal								0.04						
1749	*(E,E)‐*2,4‐Dodecadienal											0.46			
**Ketones**		**0.59**	**5.57**	**3.55**	**0.47**	**0.43**	**1.11**	**0.37**	**7.18**	**0.43**	**1.32**	**22.72**	**1.17**	**0.26**	**2.45**
899	Acetol				0.33	0.26	0.35	0.37	1.88	0.43	0.21		0.97	0.23	1.25
1010	1‐Penten‐3‐one											0.60			
1040	3‐Hexanone													0.03	
1134	*(E)‐*5‐Methyl‐4‐hepten‐3‐one											0.12			
1333	6‐Methyl‐5‐hepten‐2‐one		1.99	1.05		0.17					1.11				
1384	1‐Hydroxy‐2‐butanone											0.33			
1395	2‐Nonanone				0.05										
1420	*(E)‐*3‐Octen‐2‐one		0.08	0.08					0.63			0.46			
1544	*(E,Z)‐*3,5‐Octadien‐2‐one		2.48	1.81					2.53			17.86			1.20
1602	*(E,E)‐*3,5‐Octadien‐2‐one	0.25	1.02	0.61	0.09				2.05			2.99			
1613	6‐Methyl‐3,5‐heptadiene‐2‐one	0.34													
1626	2‐Cyclopentene‐1,4‐dione						0.76								
1690	2‐Methyl‐3‐octanone											0.36			
1714	3,4,5,5‐Tetramethyl‐2‐cyclopenten‐1‐one								0.10						
1817	2‐*t*‐Butyl‐6‐methyl‐5‐(3‐methylbutyl)[1.3]dioxan‐4‐one												0.20		
**Esters**		**16.22**	**20.88**	**17.70**	**18.23**	**40.45**	**45.46**	**32.46**	**7.68**	**7.32**	**28.89**	**4.09**	**17.07**	**32.96**	**14.46**
898	Methyl acetate	1.77	1.08	1.15											
922	Ethyl Acetate	0.98	1.15	1.47	0.59	0.46	0.22	0.26	0.71	0.78	0.36		2.08	0.21	2.43
955	Ethyl 2‐ethoxy‐2‐hydroxyacetate		0.40												
979	Methyl butanoate		0.42	0.15			0.14	0.15			0.08			0.10	
1017	Ethyl butanoate	0.08	0.34	0.19	0.10	0.05	0.17	0.09		0.34	0.05		0.08	0.12	
1043	Ethyl pentanoate		0.37	0.05				0.07							
1042	Ethyl valerate			0.02							0.08				
1105	Ethyl isovalerate		0.03												
1154	Butyl acrylate	0.10			0.23	0.04	0.03	0.19			0.13		0.13	0.11	0.13
1163	Methyl hexanoate	3.16	3.01	3.12	1.98	1.24	2.36	1.10	2.07	0.66	3.08	0.55	1.17	1.45	1.96
1197	Butyl butanoate				0.21			0.10						0.07	
1213	Ethyl hexanoate	3.51	4.20	5.50	3.17	2.37	2.82	1.90	0.92	0.90	4.20		2.02	2.71	1.94
1242	Methyl 9‐oxononanoate							0.09							
1274	Methyl heptanoate	0.08	0.18	0.14	0.11	0.11	0.28	0.18	0.10		0.10		0.15	0.15	0.08
1307	2‐Ethylbutyl acetate								0.09						
1319	Propyl trichloroacetate	0.45	0.36	0.24	0.59	0.12	0.34	0.46	0.19	0.28	0.66	0.43	0.93	0.46	0.23
1326	Ethyl heptanoate	0.09	0.19	0.31	0.17	0.24	0.27	0.33	0.02		0.24		0.24	0.28	0.08
1336	Methyl trichloroacetate	2.35			3.37		1.61	2.02	1.13	1.27		1.25	2.98	1.69	1.58
1349	Ethyl trichloroacetate	0.96	0.54	0.39	1.12	0.12	0.55	0.93	0.48	0.43	0.45	0.85	1.32	0.77	0.45
1389	Methyl octanoate	0.90	3.95	1.91	2.82	9.50	7.74	5.21	0.57	1.10	2.97	0.54	1.28	4.61	1.58
1440	Ethyl octanoate				2.98	16.78	7.04	5.83		0.91	4.74		1.32	7.30	1.08
1469	Methoxyacetic acid, 6‐ethyl‐3‐octyl ester														0.15
1492	2‐Ethylhexyl acrylate		0.30	0.30											
1502	Methyl nonanoate	0.33	0.35	0.13	0.17	1.18	3.55	1.72	1.07		0.70	0.11	0.90	2.33	0.11
1549	Ethyl nonanoate		0.19			1.03	4.39	1.17			1.13		0.95	3.43	
1574	Methoxypropyl acetate								0.17						
1603	Ethyl 8‐nonenoate												0.50		
1611	Methyl decanoate		1.03	1.20		1.99	4.48	2.95			3.43	0.06	0.56	2.62	0.26
1611	Methyl‐10‐undecanoate									0.39					
1632	Ethyl ketovalerate										0.16				
1656	Ethyl decanoate		1.65	1.04		1.47	6.74	2.55			2.14	0.25		4.09	0.13
1664	Acetopropyl acetate					0.13									
1683	Methyl isovalerate	1.00													1.98
1694	Vinyl hexanoate														0.06
1723	Ethyl undecanoate										0.72				
1728	Heptyl cyclobutanecarboxylate							0.40							
1749	Octyl acetate					1.44									
1819	Methyl laurate		0.46	0.32	0.16	0.67	0.67	2.15						0.24	
1824	Hexanoic acid, 2,2‐dimethylpropyl ester														0.24
1858	Ethyl laurate		0.54			0.81	0.80	1.50							
1885	3‐Hydroxy‐2,4,4‐trimethylpentyl isobutyrate									0.15					
1911	Nonyl acetate					0.60									
1928	Ethyl hexadecyl carbonate										1.16				
1949	*(Z)‐*Butanoic acid, 3‐hexenyl ester	0.45			0.31						0.21				
2015	Methyl myristate				0.13		0.09	0.31			0.13				
2203	Methyl palmitate		0.13	0.07		0.09	0.64	0.42	0.15	0.10	0.76	0.05		0.20	
2239	Ethyl palmitate						0.53	0.22			0.58				
2204	Methyl 8‐nonenoate												0.44		
2305	1‐Ethylpentyl acetate							0.18							
**Acids**		**48.68**	**44.37**	**50.58**	**26.27**	**40.46**	**18.08**	**15.55**	**40.00**	**32.23**	**14.87**	**19.94**	**16.90**	**33.12**	**45.97**
1444	Acetic acid	33.12	20.89	23.45	11.54	3.02	3.68	4.97	27.81	9.98	4.51	13.65	10.65	9.57	11.76
1544	Propanoic acid	2.52								1.41					
1575	2‐Methylpropanoic acid	0.14	0.18	0.13						0.07		0.07			0.08
1638	Butanoic acid	0.79	3.17	1.75	0.66	0.65	0.84	0.74		4.39		0.08		2.83	0.39
1678	Isovaleric acid		3.61	4.72						3.28		0.76			
1685	4‐Methylpentanoic acid							1.69							
1725	2‐Ethylbutanoic acid		0.40												
1727	Diethylacetic acid											0.57			
1749	Pentanoic acid	0.96	2.00	0.57	0.44		0.33	0.23	0.80	1.37	0.16			0.57	0.79
1793	*(E)‐*2‐Methyl‐2‐butenoic acid									0.28					
1808	3‐Methylpentanoic acid			0.06											7.49
1852	Hexanoic acid	9.17	7.44	12.91	7.98	11.33	5.05	1.80	8.10	4.72	5.68	3.46	2.80	8.20	13.85
1892	2‐Pentenoic acid	0.05													
1953	Heptanoic acid		0.43	0.20	0.20	0.42	0.28	0.34	0.35		0.09		0.44	0.52	0.53
1956	*(E)‐*3‐Hexenoic acid								0.11			0.06			
2051	Octanoic Acid	1.08	5.15	4.03	4.74	19.68	5.04	3.95	1.12	5.78	3.46	1.05	1.41	7.68	10.26
2148	Nonanoic acid	0.52	0.31	0.32	0.37	2.52	1.68	1.04	1.58	0.37	0.49	0.18	1.07	2.26	0.36
2240	Decanoic acid	0.33	0.72	2.44	0.34	2.84	1.18	0.79	0.13	0.58	0.48	0.06	0.53	1.49	0.46
2297	Undecanoic acid		0.07												
**Lactones**		**0.96**			**1.09**	**0.46**			**2.55**	**0.24**	**0.15**	**0.32**	**1.28**	**0.94**	
1655	*γ*‐Valerolactone				0.43										
1656	*γ*‐Pentalactone	0.30							1.02	0.24					
1677	*γ*‐Butyrolactone					0.46			1.42			0.32		0.72	
1680	Hexanoic acid, 5‐hydroxy‐3‐methyl‐, *δ*‐lactone												1.09		
1714	*γ‐*Vinyl‐*γ‐*valerolactone												0.19		
1725	*β‐*Angelica lactone	0.66			0.66									0.20	
2061	*γ‐*Nonalactone								0.11					0.02	
2185	2‐Hydroxy‐*γ*‐butyrolactone										0.15				
**Hydrocarbons**		**3.83**	**2.24**	**2.60**	**4.78**	**1.23**	**15.58**	**10.61**	**1.10**	**1.81**	**3.93**	**6.13**	**8.72**	**4.08**	**4.54**
857	2,2,3‐Trimethylbutane	1.78	0.43	0.20	1.64	0.46	0.26	1.02	0.17	0.81	0.32	1.02	1.58	0.54	1.47
889	Nonane		0.19		0.14			0.28	0.16	0.12	0.14			0.21	0.08
909	*(E)‐*2‐Octene											0.10			
928	2,4‐Dimethylheptane							0.17		0.11	0.17	0.05		0.12	
972	2,2,4,6,6‐Pentamethylheptane	0.64	0.18	0.12	0.46	0.14	0.08	0.16	0.21	0.24	0.16		0.10	0.09	0.46
991	Decane	0.28	0.35	0.33	0.37	0.14	0.25	0.47	0.16	0.32	0.75	0.21	0.76	0.30	0.27
1034	2,3,7‐Trimethyloctane		0.07									0.03			
1072	Undecane	0.22	0.28	0.19	0.25	0.11	0.13	0.34			0.38		0.34	0.19	0.20
1115	2‐Methyl‐2‐pentene											1.52			
1228	(1‐Methylethylidene)‐cyclohexane											0.05			
1240	*(Z)‐*3‐Tetradecene			0.11											
1240	2,6,11‐Trimethyldodecane								0.17						
1285	*(Z)‐*3‐Decen‐1‐yne											0.64			
1293	Tridecane	0.53	0.23	0.34	0.90	0.27	0.44	1.01			0.89	0.10	0.70	0.40	
1297	3,5,5‐Trimethyl‐1‐hexene											0.16			
1370	2‐Methyltetradecane				0.08										
1408	Tetradecane						0.45								
1485	3‐Ethyl‐1,4‐hexadiene											2.12			
1516	Pentadecane				0.29		11.99	1.12			0.48		0.42	0.72	0.28
1564	1‐Pentadecene							3.72							
1722	Heptadecane						0.15	0.25	0.04				0.22	0.15	
1741	1‐Tetradecen‐3‐yne			0.26											
1742	*(E)‐*9‐Eicosene				0.25			0.79							
1798	2,3‐Dimethyl‐2‐pentene				0.03										
1914	Nonadecane	0.22	0.28	0.92	0.08		1.14	0.41	0.19	0.13	0.24	0.07	3.26	0.59	0.59
2006	5‐Butylnonane												0.18		
2012	Eicosane														0.14
2098	Heneicosane	0.16	0.17	0.15	0.29	0.12	0.50	0.71		0.09	0.20	0.05	0.73	0.38	0.96
2276	Tricosane		0.06				0.19	0.14			0.19		0.43	0.38	0.10
**Others**				**0.38**		**0.28**						**0.21**			
1105	1‐Chloro‐2‐methylpropane			0.38											
1120	1,9‐Dichlorononane											0.21			
1511	1‐Chlorohexadecane					0.28									
**Unknown compounds**		**1.38**	**1.23**	**0.85**	**3.1**	**1.25**	**1.42**	**1.01**	**0.25**	**1.22**	**3.61**	**4.73**	**4.31**	**1.59**	**2.74**

*Note:* CN: musk thistle; CI: rock‐rose; CV: traveler's joy; FV: dropwort; GT: honey locust; HA: sunflower; PR: red poppy; PT: phacelia; PA: sweet cherry; RF: wild blackberry; TO: dandelion.

^a^ BN: rapeseed.

**FIGURE 2:**
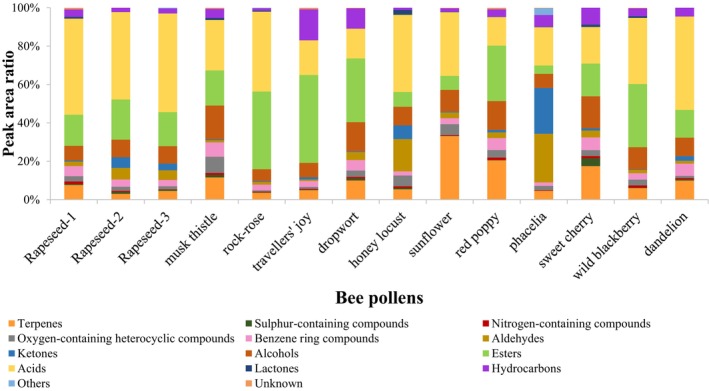
The contribution of the chemical classes to total aroma in bee pollen samples.

The number of VOCs present in bee pollens ranged between 75 and 101. The headspace of the honey locust (GT) exhibited the least, while those of the red poppy's (PR) included the most volatile compounds. Concerning the total peak area of the odorants, pollens from rock‐rose, honey locust and phacelia comprised aroma components in the highest amount. The lowest total peak area was observed in the pollen of musk thistle. The results of our experiment demonstrated the presence of 19 common odorants in the pollens. Figure 2 depicts the peak area ratio of the individual chemical groups in the total aroma.

The principal volatile organic compounds identified in the examined bee pollens were acetic acid, octanoic acid, α‐pinene and 3,5‐octadien‐2‐one. In the majority of samples, fatty acids (in particular, acetic acid, hexanoic acid and octanoic acid) constituted the primary volatiles (14.87%–50.58%), whereas in certain samples, esters (predominantly methyl octanoate, ethyl octanoate, methyl and ethyl hexanoate) were the most prevalent odorants. In Polish bee pollen (Starowicz et al. 2021), acids constituted the second most dominant volatile (11.8%), after alkanes (65.5%). Similarly, the predominant fatty acids in Italian ivy pollen were acetic acid and hexanoic acid, as observed in our own results (Filannino et al. 2021). Fatty acids can be present in pollen in the form of triglycerides or in free form (Cagliari et al. 2011) and their source could be the pollenkitt (Dobson and Bergström 2000; Piskorski, Kroder, and Dorn 2011).

Additionally, esters were identified as a significant component of bee pollens (4.09%–45.46%). The most intense esters present in the samples were ethyl octanoate (0.91%–16.78%) and methyl octanoate (0.54%–9.50%). In Turkish bee pollen, esters were also prevalent volatiles, representing over half of the total aroma components (Keskin and Özkök 2020). A similar result was observed in Brazilian pollen samples, whereby the application of different sampling methods led to the identification of a variety of esters within the samples (Lima Neto et al. 2017). Esters are usually synthesized by the reactions between alcohols and carboxylic acids or between alcohols and acyl‐CoA molecules. These processes are catalyzed by microbial esterase or acyltransferase enzymes (Liu, Holland, and Crow 2004). The majority of esters exhibit a fruity flavor profile.

In terms of their numbers, terpenoid compounds were the most prevalent VOCs in bee pollen, with a total of 68 distinct terpenes identified in the samples. Terpenes are usually indicators of plant origin in apiculture products (Aronne et al. 2014; de Oliveira et al. 2021; Jerković et al. 2014; Kasote, Bankova, and Viljoen 2022; Machado et al. 2020; Manyi‐Loh, Ndip, and Clarke 2011; Prđun et al. 2021; Pujirahayu, Suzuki, and Katayama 2019; Starowicz et al. 2021; Tedesco et al. 2022) and appear in great variety. A number of terpenoids arise during the oxidative degradation of carotenoids (Karabagias et al. 2021). Several mono‐, sesquiterpenes and other terpene derivatives were identified as minor compounds in the bee pollens of different botanical origin. In the majority of pollens, peak area% of terpenes accounted for less than 12%, and their number was between 8 and 18. The volatile fraction of sunflower (33.14%), red poppy (19.06%) and sweet cherry pollens (17.59%) were the wealthiest in terpenes in terms of their peak area ratio. The most abundant volatile group in sunflower pollen was that of terpenes (Figure 2). The sample exhibited an exceptionally high peak area ratio (19.60%) of *α*‐pinene, which is in accordance with the findings reported by Bertoli et al. (2011). The number of terpenoids were also the highest (26, 20 and 23) in the above mentioned three samples. D‐limonene and geranyl acetone were the common terpene constituents in our bee pollens. Investigating the volatile composition of different plant parts of *Citrus limon* , Flamini, Tebano, and Cioni (2007) identified limonene in every tissues except for the pollen. Some research works (Aggarwal et al. 2002; Chalchat et al. 2000; Dorman and Deans 2000; Van Vuuren and Viljoen 2007) has attributed antimicrobial effects to this monoterpene compound. Additionally, linalool and its derivatives (comprising two isomers of lilac aldehyde and four isomers of lilac alcohol) were identified in the volatile fraction of certain samples. Mono‐ and sesquiterpenes were also dominated (77%) in the pollen of Italian *Citrus limon* plant with *trans*‐nerolidol as the major component (Flamini, Tebano, and Cioni 2007). *Cannabis sativa* pollens were also rich in high‐intensity terpenoid compounds with *β*‐myrcene, trans‐*β*‐ocimene and terpinolene as the main volatiles (Rothschild, Bergström, and Wängberg 2005). Bee pollen samples from France were found to be rich in monoterpenes like *α*‐ and *β*‐pinene, sabinene and camphene (Collin et al. 1995). In comparison to our samples, Italian bee pollen derived from ivy ( *Hedera helix* L.) exhibited a markedly reduced terpenoid content, with only four components detected in trace amounts (Filannino et al. 2021). A remarkable number of characteristic terpenoids have been identified in our sunflower pollen. These were *α*‐terpinene (0.52%), *β*‐phellandrene (0.12%), *β*‐cymene (0.96%), *α*‐terpinolene (0.15%), 4,8‐dimethyl‐3,7‐nonadien‐2‐ol (0.02%), farnesane (0.07%), tetrahydrolinalool (0.03%), *δ*‐cadinol (1.01%), 4‐terpineol (0.16%), 1‐menthol (0.11%), *β*‐chamigrene (0.76%), perilla aldehyde (0.32%), *cis*‐verbenol (0.16%) and dihydro‐carveol (0.19%). Linalool derivatives lilac aldehyde A, B and C as well as lilac alcohol A, B, C and D, together with *β*‐cyclocitral were among the unique fragrance constituents of sweet cherry pollen. Red poppy (ethyl iso‐allocholate, *β*‐citral, *trans*‐2‐pinanol, geranyl acetate, citral), dandelion (borneol, nerolidol), phacelia (isothujol, epoxy‐linalool oxide), honey locust (*α*‐copaene, *γ*‐muurolene), rapeseed‐3 (isoborneol) and traveler's joy (verbenone) pollens also contained specific terpenoid components. In *Cannabis sativa* pollen longifolene, menthol, ipsdienol and *β*‐cedrene were identified as characteristic volatiles of this plant (Rothschild, Bergström, and Wängberg 2005). Terpenoids were also abundant in *Rosa rugosa* pollen with geranyl acetone, geranial and geranyl acetate as the major odorants within this group (Dobson et al. 1987).

Sulfur‐containing VOCs have also appeared in the volatile fraction of every bee pollen (0.07%–4.07%). Sweet cherry pollen exhibited the greatest diversity of sulfur compounds, with dimethyl sulfide (2.85%) and dimethyl sulfoxide (1.06%) representing the most intense constituents within this group (Figure 2). These aroma components are usually volatile decomposition products of sulfur‐containing amino acids like S‐methylmethionine. French pollen samples with an unpleasant odor reminiscent of cooked onions were found to contain both dimethyl sulfide and dimethyl disulfide (Collin et al. 1995).

Additionally, nitrogen‐containing volatiles have been identified in the pollen samples, with a relatively low ratio (0.20%–1.32%). These volatile components are supposed to form during the breakdown of amino acids. The components are classified into various chemical groups, including isocyanides, pyrazoles, pyrrolidines, nitriles and pyrroles as well.

Benzene derivatives were identified in the samples at minor percentages (1.82%–7.44%). Members of this compound group are mostly formed from amino acids (e.g., phenylalanine) during enzymatic or non‐enzymatic processes (Prđun et al. 2021). Benzaldehyde was present with significant peaks in all samples except for dropwort pollen, and three benzenoid compound (toluene, ethylbenzene and styrene) have appeared in all pollen samples. Among these common volatiles, styrene had been identified previously (Cao, Sparling, and Dabeka 2016; Cao et al. 2018; Genualdi, Nyman, and Begley 2014; Guazzotti et al. 2022; Khaksar and Ghazi‐Khansari 2009; Kaškonienė, Kaškonas, and Maruška, 2015; López et al. 2008; Schwarz, Boitz, and Methner 2012; Silva et al. 2000; Starowicz et al. 2021; Steele et al. 1994) in several food products like meats, dairy products, cereals, vegetables, fruits, pollen, fats and oils, beverages, baby food and fast food. Polish bee pollen sample has also contained this constituent, it accounted for 1% of the total volatile components (Starowicz et al. 2021). In Lithuanian bee pollen, Kaškonienė, Kaškonas, and Maruška (2015) also identified styrene in quite high peak ratio. The sample was found to contain this compound, representing between 19.6% and 27.0% peak area ratios of total volatiles. The bee pollens examined in this study exhibited low ratios (0.20%–0.83%) of styrene. There are several explanations for the origin of styrene in food. Most likely source of this volatile is the packaging material (Ajaj et al. 2021; Genualdi, Nyman, and Begley 2014; Guazzotti et al. 2022; Kaškonienė, Kaškonas, and Maruška, 2015; Kubica et al. 2018; Pilevar et al. 2019; Sadighara et al. 2022; Tang, Hemm, and Eisenbrand 2000), as styrene is used in the production of several types of plastics (polystyrene, polyamide, acrylonitrile‐butadiene‐styrene) and styrene in polystyrene has the ability to migrate into food (Cao, Sparling, and Dabeka 2016). Styrene may also be a biodegradation product of some aromatic substances (e.g., cinnamic acid, cinnamic aldehyde, cinnamyl acetate), so high levels are usually detected in cinnamon (Cao, Sparling, and Dabeka 2016; Cao et al. 2018; Steele et al. 1994; Tang, Hemm, and Eisenbrand 2000). As styrene was identified in all pollen samples, the source is likely to be the packaging material, as the pollen was in direct contact with the plastic bag. Similarly to styrene, toluene also may be an external (environmental) pollutant in bee pollen (Karabagias et al. 2021).

Alcohols (6.21%–15.10%), aldehydes (0.83%–25.30%) and ketones (0.26%–22.72%) are notable volatile compounds present in bee pollens. Within the compound group of alcohols, ethanol was a common VOC present in all samples. This odorant was the most prevalent alcohol in bee pollen (2.06%–14.61%). While alcohols can serve as significant fragrance compounds in food products, ethanol does not typically contribute to the overall flavor of foods through its neutral odor (Berger 2007). Ethanol is produced by the process of microbial glycolysis and is considered a landmark compound of environmental pollution and fermentation of fruits and vegetables (Ni et al. 2023). Volatile alcohols were present in the headspace of raw ivy pollen and their amount is highly increased during fermentation (Filannino et al. 2021). Alcohols can be produced from the reduction of aldehydes, while the precursors of branched alcohols are branched amino acids like leucine, isoleucine and valine (Ardö 2006).

Aldehydes are important intermediates in lipid oxidation (Ni et al. 2023), and due to their relatively low perception threshold values, they are key volatiles in the flavor of a number of foods (Bi et al. 2024; Lima et al. 2023; Machado et al. 2020; Wang et al. 2023; Yu et al. 2022). The pollen of phacelia was found to be particularly rich in aldehydes, with the peak area of the 17 compounds accounting for 25.30% of the total aroma. The volatile fraction of all pollen samples exhibited the presence of hexanal and nonanal (0.20%–11.83%), which are oxidation products of linoleic and oleic acids. These aldehydes were also identified in Lithuanian (Kaškonienė, Kaškonas, and Maruška, 2015) and German (Jürgens and Dötterl 2004) bee pollens in relatively high amount. According to Dobson and Bergström (2000), the presence of nonanal in the volatile fraction of pollens may have a defensive role against pollen‐feeding animals. In the case of bee‐collected pollen, some headspace volatiles may derive from the pollinators as well. For example, aldehydes with longer chain length (from C9 to C17) were reported as the principal headspace volatiles of the foraging bees *Apis mellifera* (Schmitt et al. 2007). Beyond the cuticles of the honeybees, the source of these aldehydes might be the comb wax as well (Blum et al. 1988). These odorants may transfer from the headspace of the bee to the lipids in pollenkitt and appear as pollen VOCs (Prđun et al. 2021). Volatile aldehydes and alcohols are the characteristic products of the lipoxygenase (LOX) pathway and autoxidation, but they can be the intermediates of amino acid degradation as well (Berger 2007). In Greek pollen samples aldehydes were identified as the dominant volatile compound group, representing 48.47 area% of the total volatile compounds. The major constituents within this class were hexanal and heptanal (Karabagias et al. 2021). The volatile fraction of Italian ivy ( *Hedera helix* L.) pollen was also predominated by aldehydes (30% of total VOC), mostly by hexanal (Filannino et al. 2021). In addition to lipid‐derived aldehydes, the bee pollen samples analyzed in this study also contained aldehydes that are formed during the breakdown of amino acids (Strecker aldehydes).

Ketones were identified in smaller numbers (1–7) in bee pollen, with 3,5‐octadien‐2‐one isomers and 6‐methyl‐5‐hepten‐2‐one as the dominant VOCs within this group. In the research conducted by Filannino et al. (2021), Italian ivy pollen was also found to contain these ketones in the greatest abundance. In addition to aldehydes, phacelia pollen exhibited a markedly elevated abundance of ketones relative to other samples. The seven ketones identified in the sample collectively constituted 22.72% of the total peak area. Long‐chain ketones (2‐undecanone, 2‐tridecanone, 2‐pentadecanone) were also found at high levels in *Rosa rugosa* pollen (Dobson et al. 1987). In this latter study, about half of the aroma compounds found in pollen were also identified in the pollenkitt. Ketones can be produced by the oxidation of fatty acids. Numerous ketones (mostly *α*‐methyl ketones) have been shown to have a deterrent or even toxic effect on certain insects and pathogens, and some of them show antifungal activity (Borries et al. 2019; Dobson and Bergström 2000; Innocent, Gikonyo, and Nkunya 2008; Marr and Tang 1992; Zhu et al. 2018). The volatile fraction of musk thistle pollen was found to contain 2‐nonanone, a ketone with the potential to exert a particular effect.

In the volatile fraction of some pollen samples, the presence of lactones has also been identified (0.15%–2.55%). In the production of lactones, proteins and fatty acids may be involved as precursors (Aydin 2017; Lee et al. 2018). The predominant compounds identified in pollen samples are *γ*‐lactones, including *γ*‐valerolactone, *γ*‐pentalactone, *γ*‐butyrolactone, *γ‐*vinyl‐*γ‐*valerolactone, *γ‐*nonalactone, and 2‐hydroxy‐*γ*‐butyrolactone.

The pollen samples exhibited a considerable diversity of hydrocarbon profiles, although their peak ratios were relatively low (1.10%–15.58%, Figure 2). Among hydrocarbons, 2,2,3‐trimethylbutane and decane were present in all pollen samples, while the intensities of tridecane and nonadecane were the most abundant in the majority of the pollens. The principal component of traveler's joy pollen was pentadecane (11.99%). This alkane hydrocarbon also dominated in *Pulsatilla* species ( *P. rubra* and *P. vulgaris* ) from Germany (Jürgens and Dötterl 2004). It can be assumed that this compound is a typical volatile component of plants belonging to the taxonomic tribe *Anemoneae*, as both traveler's joy ( *Clematis vitalba* ) and *Pulsatilla* species are included in this group. Various studies (Eisner, Rossini, and Eisner 2000; Jürgens and Dötterl 2004; Krall et al. 1999; Zinner 1986) have shown that pentadecane can play a protective role against herbivorous insects and is often used against predators of various insect taxa. In Polish bee pollen, Starowicz et al. (2021) found alkanes as the main compound group (65.5% of total volatiles). Dodecane, tridecane and undecane were the leading constituents in some bee pollens from China, Latvia and Lithuania as well (Kaškonienė, Ruočkuvienė, et al. 2015). Long‐chain hydrocarbons deriving from fatty acid degradation have also dominated the volatile fraction of Italian *Citrus deliciosa* pollen, 1‐heptadecene and n‐heptadecane provided more than half (56.3%) of the peak area of all volatile compounds (Flamini, Cioni, and Morelli 2003). Aliphatic hydrocarbons, mostly alkanes with high carbon number (C16 <) are considered to play an important role in kin recognition (Arnold et al. 1996; Arnold, Quenet, and Masson 2000; Breed and Stiller 1992; Fröhlich, Riederer, and Tautz 2001; Getz and Page Jr. 1991) and may be the constituents of the cuticle of honeybees (Fröhlich, Riederer, and Tautz 2001; Martin et al. 2001; Schmitt et al. 2007).

The differences in the aroma profile of pollens reflected the diversity of their botanical origin. In addition to the source plant, the geographical origin, climatic conditions and bee species also exert a significant influence on the formation of the volatile profile of bee pollen.

**FIGURE 3:**
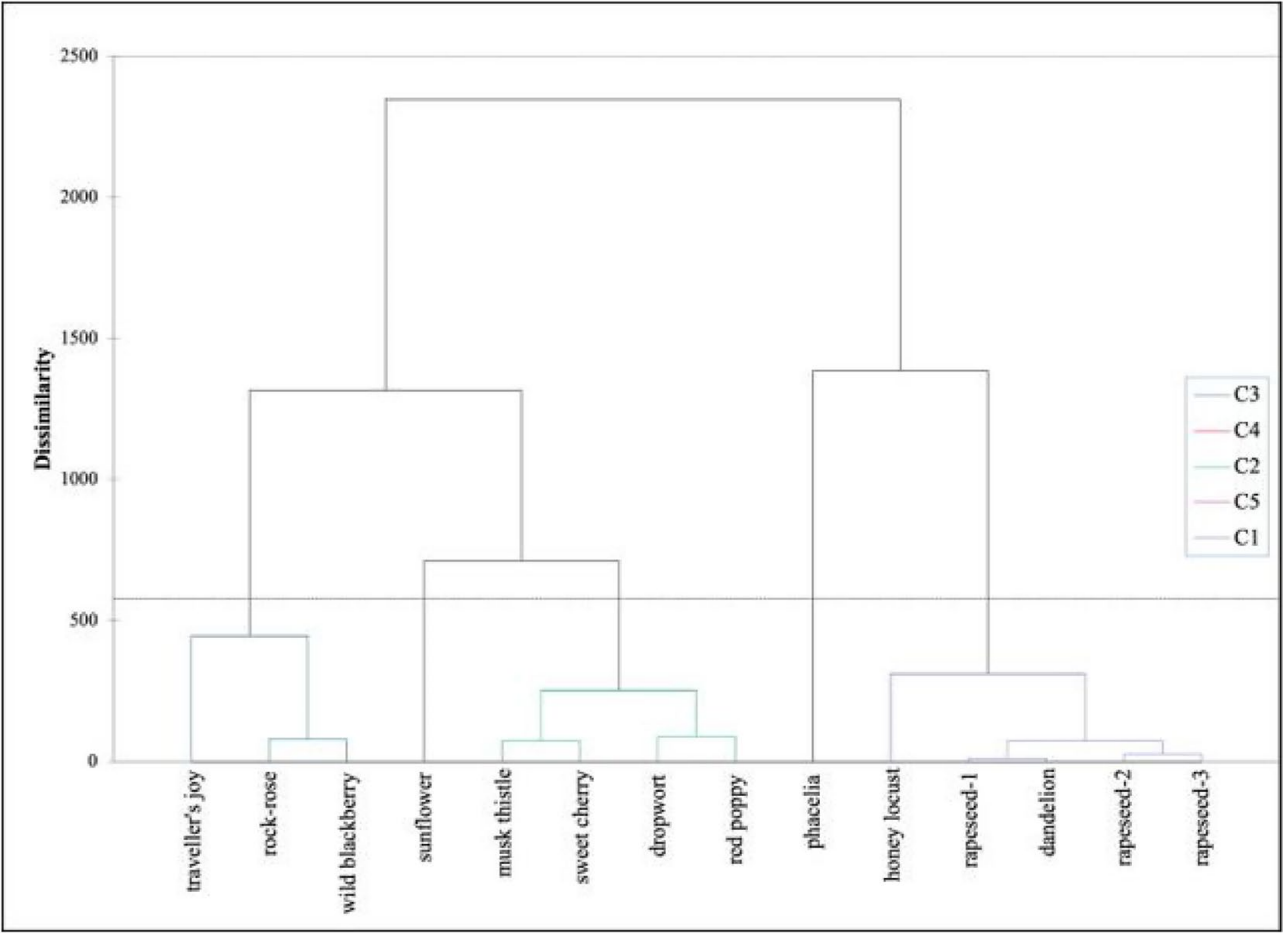
Dendrogram of agglomerative hierarchical clustering of pollens (Ward's method, Euclidean distance). The horizontal line indicates the boundary of the clusters.

An important result of the agglomerative cluster analysis (Ward's method, Euclidean distance) is the dendrogram, which shows the differences and similarities of pollen samples aroma groups. Pollen that belong to one cluster are similar, those that belong to different clusters are different. The more distant a pollen (cluster member) is from another pollen the less similar they are. Based on the cluster indices (Silhouette index, Calinski index and Harabas index), it is appropriate to distinguish five pollen groups (cluster groups) (Figure 3).

The five clusters were found to be significantly different (*p* < 0.05) in terpenes, benzene ring compounds, aldehydes, ketones, alcohols, esters, acids, and components classified as “others”.

#### 3.3.2 Odor‐Active Compounds in the Pollen Samples

The volatile components of the bee pollen samples were characterized by gas chromatography–olfactometry (GC‐O). The objective of this analysis was to identify the aroma‐active zones and constituents through a sensory evaluation of the eluate from the GC column. The aroma descriptions of the odor‐active compounds are provided in Table 3.

**TABLE 3:** Odor characteristics of the VOCs perceived in the pollen samples.

RI^a^	Compound	Aroma description^b^	Aroma intensity^c^													
			BN‐1	BN‐2	BN‐3	CN	CI	CV	FV	GT	HA	PR	PT	PA	RF	TO
**Terpenoids**																
1007	*α*‐Pinene	Fruity														2
1043	Camphene	Green									1					
1093	Sabinene	Green, weed											2			
1194	Eucalyptol	Menthol, camphor			1											2
1228	*γ*‐Terpinene	Herbal									1	1				
1232	Ocimene	Unknown	1									1				
1295	4,8‐Dimethyl‐3,7‐Nonadien‐2‐ol	Mushroom									2					
1363	Phytane	Sweet, baked, nutty, roasted nut, caramel				3		3				2		3	3	3
1437	Tetrahydrolinalool	Flower									1					
1471	Dihydromyrcenol	Green, vegetable												1		
1513	*α*‐Copaene	Dust								2						
1553	Isothujol	Fruity											1			
1555	Linalool	Flower										2				
1618	Lilac aldehyde C	Sour, vegetable												2		
1621	Hotrienol	Sour, baked, herbal, cucumber, vegetable, fruity, raw	2			3	3		2	2		2	3			3
1624	*δ*‐Cadinol	Herbal									2					
1625	Caryophyllene	Baked, herbal				3	3									
1630	4‐Terpineol	Herbal									2					
1658	1‐Menthol	Honey, sweet									2					
1740	Geranyl acetate	Sour, herbal										2				
1752	Lilac alcohol A	Warm fruit, jam												1		
1761	Verbenone	Sour, herbal						2								
1778	D‐Carvone	Herbal, quince					1								1	
1794	*α‐*Curcumene	Baked, flower, fruity, plum, exotic, meat soup powder	2				2	2	3			1		2	1	
1802	Artemisia ketone	Fruity			2											
1849	Lilac alcohol D	Fruity, plum, red fruit												2		
1874	Geranyl acetone	Flower									1					
1883	*α‐*Ionone	Flower, pleasant, perfume, sweet, herbal			1			1						1	1	1
1969	*β*‐Ionone	Herbal, fruity, flower, perfume						3	3					1		3
2012	Caryophyllene oxide	Flower				1										
2022	*β*‐Ionone epoxide	Flower						1						1	1	
2041	Nerolidol	Baked														2
**Sulfur‐containing compounds**																
878	Dimethyl sulfide	Sunflower seed, green		1					1					1		
1383	Dipropyl disulfide	Raw, green, herbal, vegetable, baked, sweet, caramel	3			3	2		2		2	1		1	1	2
1641	Dimethyl Sulfoxide	Unpleasant						1								
**Nitrogen‐containing compounds**																
1001	Methyl isocyanide	Sweet	1													
1955	Benzyl nitrile	Rubber		1												
2088	2‐Pyrrolidinone	Herbal														1
**Oxygen‐containing heterocycles**																
1347	Tetramethyloxirane	Baked, sweet											3			
1485	Furfural	Cooked vegetables, boiled potato, herbal, sweet, pollen, cheese, earthy		2	2	2		1	2	1	1	3			1	
1609	5‐Methylfurfural	Green, cucumber				3										
1676	2‐Furanmethanol	Flower													1	
1807	5‐Ethyl‐2(5H)‐furanone	Lemon, citrus, fruity, sour, berry	2												2	
1891	Furaneol	Baked		1												
2264	2,3‐Dihydro‐3,5‐dihydroxy‐6‐methyl‐4H‐pyran‐4‐one	Cheese, black pepper		1						3						
**Benzenoids**																
1026	Toluene	Fruity, apple, sweet, vanilla, green, flower	3		2	2	1	2	3	1		2		1		1
1104	Ethylbenzene	Herbal, sweet, apple, fruity								2	1	2		1	1	1
1116	*o*‐Xylene	Fruity, apple		2	2											
1118	*p*‐Xylene	Sweet, fruity	2			1	1	2	1							
1251	Styrene	Plastic												1		
1258	2‐Ethyl‐1,4‐dimethylbenzene	Unknown														1
1434	1,3‐Dichlorobenzene	Fruity, apple, green						2	1			2		1		
1561	Benzaldehyde	Plastic, cardboard									2		3	1		
1693	Acetophenone	Roasted, sweet, baked, nutty, instant meat soup, sour	1			1	1					2		2		
1896	Benzyl Alcohol	Unknown													1	
1931	Phenylethyl Alcohol	Flower	1	1						1			2		1	2
1968	p‐Methylguaiacol	Flower										2				
2163	Eugenol	Sweet biscuit			1											
**Alcohols**																
942	4‐Methoxy‐1‐butanol	Herbal							1							
946	Ethyl alcohol	Herbal								1						
1047	2‐Methylpentanol	Fruity, apple		1												
1130	1‐Penten‐3‐ol	Green, vegetal											2			
1228	1‐Pentanol	Green, herbal		2												
1307	*(Z)‐*2‐Penten‐1‐ol	Mushroom		2									3			
1378	*(Z)‐*3‐Hexen‐1‐ol	Unknown, wet, rain		2	2											
1405	*(E)‐*2‐Nonen‐1‐ol	Earthy									1					1
1492	2‐Ethylhexanol	Cooked vegetables									1					
1521	2‐Decanol	Unknown										1				
1543	1‐(2‐Methoxypropoxy)‐2‐propanol	Spicy													1	
1552	3‐Methyl‐2‐hexanol	Flower, spicy, herbal		1	1				1		2					
1553	2,3‐Butanediol	Flower				3				1						
1564	1‐Octanol	Plastic, textile, herbal		2	2		3		1	3	2	3				
1620	2,6‐Dimethyl‐3,7‐octadiene‐2,6‐diol	Cooked vegetables, cucumber, green		2	2											
1634	2,6‐Dimethylcyclohexanol	Baked												1		
1677	2‐Ethyl‐1‐hexanol	Honey, sweet									2					
1687	1‐[2‐(Allyloxy)‐1‐methylethoxy]‐2‐propanol	Unpleasant, sour, rotten			3											
1760	*(E,Z)‐*3,6‐Nonadien‐1‐ol	Sour			1											
1812	2‐Ethyl‐2‐hexen‐1‐ol	Fruity, baked apple											2			
**Aldehydes**																
868	Acetaldehyde	Unpleasant, sulfury, sour			1								2			
887	Propanal	Herbal, green											1			
939	3‐Methylbutanal	Green, herbal	1	1	2							1	1			
980	Pentanal	Herbal, sweet, sour	3							3			3			
1040	2‐Butenal	Fruity, apple, herbal		1				2								
1062	Hexanal	Green, grass, vegetal, apple			2				1	2		1	3	1	1	
1146	2‐Methyl‐2‐pentenal	Fruity		1												
1210	*(E)‐*2‐Hexenal	Unknown											1			
1284	Octanal	Citrus, orange, lemon, lime, fruity, apple, tutti‐frutti		1	2			1	2	3			3			
1331	*(Z)‐*2‐Heptenal	Baked, sweet											1			
1402	Nonanal	Cabbage, herbal, sweet, baked, green, spicy, citrus, lemon, roasted meat	3	2	2	3			2	3		2	2	1		
1431	*(E,E)‐*2,4‐Hexadienal	Sweet											1			
1609	*(E,Z)‐*2,6‐Nonadienal	Fruity, raw											3			
1749	*(E,E)‐*2,4‐Dodecadienal	Baked, sweet											3			
**Ketones**																
1010	1‐Penten‐3‐one	Herbal, raw											3			
1040	3‐Hexanone	Apple													1	
1333	6‐Methyl‐5‐hepten‐2‐one	Butter, milk, sweet, instant soup, meaty					1					1				
1384	1‐Hydroxy‐2‐butanone	Green, grass											3			
1395	2‐Nonanone	Herbal, green				3										
1420	*(E)‐*3‐Octen‐2‐one	Green											1			
1602	*(E,E)‐*3,5‐Octadien‐2‐one	Green, cucumber, harsh, linden, flower				3				2			2			2
1613	6‐Methyl‐3,5‐heptadiene‐2‐one	Baked	2													
1626	2‐Cyclopentene‐1,4‐dione	Herbal, sweet						2								
**Esters**																
922	Ethyl Acetate	Sweet			1											
955	Ethyl 2‐ethoxy‐2‐hydroxyacetate	Green		1												
979	Methyl butanoate	Butter, sour, green, herbal, sweet		1	2			1	1			2				
1017	Ethyl butanoate	Rubber, plastic, sweet, caramel, green, herbal, flower, sweet, nutty, fruity	2	1	2	1	1	3	2		1	1		1	2	
1042	Ethyl valerate	Fruity, green, herbal			2							1				
1105	Ethyl isovalerate	Fruity, apple		2												
1154	Butyl acrylate	Green apple, fruity, herbal	1				1	1				1		1		2
1163	Methyl hexanoate	Fruity, apple, green, herbal	1	1	2	1		2		1		2		1	1	
1213	Ethyl hexanoate	Fruity, green apple, strawberry, berry, sweet, herbal	3	2	2	2	2	3	2	2	1	2		1	2	2
1242	Methyl 9‐oxononanoate	Unknown							1							
1307	2‐Ethylbutyl acetate	Mushroom, earthy								3						
1326	Ethyl heptanoate	Herbal, oily, sunflower seed, instant meat soup				1					1			2	1	1
1336	Methyl trichloroacetate	Herbal, oily, instant meat soup				1			1				1	2		
1349	Ethyl trichloroacetate	Baked, roasted nuts, slightly burnt, sweet, caramel	3	3	3	3	3	3	3	3	3	3	3	3	3	3
1389	Methyl octanoate	Herbal, green, roasted meat, baked, vegetable, cabbage, sweet, caramel				3	2	1	2	3	2	2			2	
1440	Ethyl octanoate	Baked, roasted seeds, green, cucumber, vegetal, herbal, unpleasant				3		2	1			3		3	2	1
1492	2‐Ethylhexyl acrylate	Unknown										3				
1549	Ethyl nonanoate	Vegetable, green, fruity, herbal					2	1				3		2	1	
1603	Ethyl 8‐nonenoate	Baked												3		
1611	Methyl decanoate	Cucumber, green, sour, vegetable, baked, fruity, raw			2		3	2	2			1	3	3	2	3
1683	Methyl isovalerate	Sour, unpleasant	3													3
1694	Vinyl hexanoate	Flower														2
1885	3‐Hydroxy‐2,4,4‐trimethylpentyl isobutyrate	Flower									1					
1911	Nonyl acetate	Flower					1									
1928	Ethyl hexadecyl carbonate	Flower, hyacinth										2				
2203	Methyl palmitate	Unpleasant, harsh, green, boiled/cooked vegetable, herbal, plastic, rubber, spicy		1				3	1	2	1	1		1	1	
2239	Ethyl palmitate	Vegetable, sour							2							
**Acids**																
1444	Acetic acid	Sour, vinegar, green, pea, baked, roasted seeds, unpleasant, vegetal, earthy, roasted coffee	3	2	2	3	3	1	1	3			2		3	1
1544	Propanoic acid	Green, weed									1					
1575	2‐Methylpropanoic acid	Cucumber									1					
1638	Butanoic acid	Cheese, sour, unpleasant		2	1		1				3				1	
1678	Isovaleric acid	Sour, fermented, unpleasant, rotten		2	3						3		2			
1749	Pentanoic acid	Sour, herbal, green, raw fruit, baked, sweet	2	2	2	2		2	2	2	1	2	3		2	2
1793	*(E)‐*2‐Methyl‐2‐butenoic acid	Honey, sweet									1					
1808	3‐Methylpentanoic acid	Fruity, sour														2
1852	Hexanoic acid	Citrus, green, sour, unpleasant, fermented, fruity	2	1	2	3	2	2	1	2	1	2			2	2
1892	2‐Pentenoic acid	Unknown	1													
1953	Heptanoic acid	Flower, perfume, spicy												1		
1956	*(E)‐*3‐Hexenoic acid	Flower								2			1			
2051	Octanoic Acid	Baked, slightly burnt, roasted, sweet		2	2	3	3	3	3	3		2	1		2	1
2148	Nonanoic acid	Spicy, perfume		1			1	1			2				1	
2240	Decanoic acid	Paint, watercolor, herbal, dill, green, unpleasant, flower		1		2	1			2	1		1	1	1	
**Lactones**																
1677	*γ*‐Butyrolactone	Green, herbal								3					2	
2061	*γ*‐Nonalactone	Coconut, sweet, almond								2					1	
**Hydrocarbons**																
857	2,2,3‐Trimethylbutane	Unknown							1							
972	2,2,4,6,6‐Pentamethylheptane	Herbal, sweet, butter, fruity	3		2	2	1	1						1		3
1034	2,3,7‐Trimethyloctane	Apple, sweet, flower		1									2			
1228	(1‐Methylethylidene)‐cyclohexane	Sweet, herbal											2			
1293	Tridecane	Mushroom					2					3	3	2	2	3
1370	2‐Methyltetradecane	Sweet, baked				3										
1485	3‐Ethyl‐1,4‐hexadiene	Sweet, raw bean, pea											2			
1798	2,3‐Dimethyl‐2‐pentene	Fruity				2										
1914	Nonadecane	Perfume, flower, fruity, herbal, biscuit		2	2									1	1	1
2276	Tricosane	Black pepper		1							1	1		1		
**Unknown compounds**																
999	Unknown	Caramel, sweet, butter		1											1	
1032	Unknown	Fruity			2										1	
1104	Unknown	Fruity, vegetal			2								2			
1241	Unknown	Green, herbal		2		1										1
1305	Unknown	Mushroom, earthy			2											
1464	Unknown	Flower														1
1487	Unknown	Herbal, sweet				2										
1519	Unknown	Unknown										1				
1555	Unknown	Plastic, rubber, herbal				2	1									1
1562	Unknown	Baked						2								
1586	Unknown	Unknown													1	
1634	Unknown	Baked	2													
1673	Unknown	Unpleasant, sour, baked				3		1				1	2			
1700	Unknown	Sweet, honey, floral, flour								2				1		2
1741	Unknown	Herbal, sour, green			2											
1762	Unknown	Quince													1	
1796	Unknown	Fruity, citrus, cooked apple		2						3		2				
1842	Unknown	Unknown											2			
1894	Unknown	Unknown								2					1	
1955	Unknown	Flower, perfume, spicy			3	2					3			1		
2019	Unknown	Fruity														1
2087	Unknown	Spicy												1	1	

^a^ Mean of RI of the samples.

^b^ Aroma impression perceived at the olfactory detection port.

^c^ Aroma intensities observed by the panelists. 1–weak; 2–middle; 3–strong. Odor characteristics perceived by at least two measurements are reported.

The majority (51.7%–69.0%) of VOCs identified in bee pollen samples by GC–MS exhibited no aroma activity, and none of the compounds displayed a characteristic pollen odor. The lowest number of aroma‐active zones (25) was identified in rapeseed‐1 pollen, while the highest number (38) was observed in rapeseed‐2, red poppy and wild blackberry pollens. In terms of the odor strength of volatile compounds, musk thistle and phacelia pollens contained the most aroma constituents with strong intensities (17 and 15, respectively). The number of identified odor zones and aroma‐active compounds showed slight differences due to the presence of multiple constituents at some fragrance‐active sites. In accordance with the aforementioned result, rapeseed‐1 pollen was found to contain the lowest number of fragrance constituents detected by the human nose (26), while red poppy and wild blackberry pollens exhibited the highest number (42) of such constituents, as identified by GC‐O. In terms of the number of VOCs, approximately one‐third of the volatile compounds possessed a discernible odor, although the remaining constituents also contribute to the overall aroma of bee pollen. The presence of toluene, ethyl butanoate, ethyl hexanoate, acetic acid, pentanoic acid, hexanoic acid and octanoid acid was identified in at least 10 pollens. As illustrated in Table 3, a total of 177 aroma‐active volatiles were perceived in the bee pollen samples, with 155 of these being confirmed by MS analysis including 32 terpenoids, 3–3 sulfur‐ and nitrogen‐containing compounds, 7 oxygen heterocycles, 13 benzenoids, 20 alcohols, 14 aldehydes, 9 ketones, 27 esters, 15 acids, 2 lactones and 10 hydrocarbons. Among the identified constituents, fatty acids were the most potent aroma‐active odorants, perceptible in the majority of the samples. These odors were predominantly medium to high in intensity and characterized by sour, green, and herbal notes. The aroma of bee pollen is the result of the combined perception of several different aroma notes, which are perceived by the olfactory system. The panelists confirmed the presence of six principal sensory characteristics, which they described as “green/sour”, “fruity”, “spicy/herbal”, “earthy/mushroom”, “sweet/baked/caramel/honey” and “floral” compounds.

The “green/sour” odor group included representatives of many different aroma constituents, such as terpenes (camphene, sabinene, dihydromyrcenol, lilac aldehyde), alcohols (1‐penten‐3‐ol, 1‐pentanol, *trans,cis*‐3,6‐nonadien‐1‐ol), aldehydes (propanal, 3‐methylbutanal, pentanal, hexanal), ketones (1‐hydroxy‐2‐butanone, 2‐nonanone, *trans*‐3‐octen‐2‐one, 3,5‐octadien‐2‐one) and acids (propanoic acid, 2‐methylpropanoic acid, pentanoic acid, hexanoic acid). Unsaturated aldehydes and alcohols with characteristic green flavor are usually breakdown products of fatty acids (Bi et al. 2024). The key sources of green, grassy aroma may be pentanal, 1‐hydroxy‐2‐butanone and 2‐nonanone because of their strong intensities, and hexanal, pentanoic acid and hexanoic acid, owing to their widespread presence in the samples.

The aroma quality of bee pollens was found to be significantly influenced by the presence of “fruity” aromas. This fragrance character might be caused by the presence of terpenes (e.g., *α*‐pinene, isothujol, *α*‐curcumene, lilac alcohol), aldehydes (e.g., 2‐butenal, 2‐methyl‐2‐pentenal, octanal) and esters (e.g., ethyl valerate, ethyl isovalerate, methyl hexanoate, ethyl hexanoate), which were identified in almost all of the samples.

“Spicy/herbal” fragrances were mainly terpenes (eucaliptol, *γ*‐terpinene, caryophyllene, *δ*‐cadinol, 4‐terpineol, geranyl acetate, verbenone, D‐carvone), but heterocyclic compounds (2‐pyrrolidinone, *γ*‐butyrolactone), alcohols (4‐methoxy‐1‐butanol, 1‐(2‐methoxypropoxy)‐2‐propanol, 3‐methyl‐2‐hexanol), aldehydes (nonanal), ketones (1‐penten‐3‐one, 2‐cyclopentene‐1,4‐dione) and acids (heptanoic acid, nonanoic acid) also possessed this kind of odor notes.

4,8‐dimethyl‐3,7‐nonadien‐2‐ol, *cis*‐2‐penten‐1‐ol, *trans*‐2‐nonen‐1‐ol, 2‐ethylbutyl acetate and tridecane contributed towards the “earthy/mushroom” odor. Mushroom‐like notes were consistently present in pollen samples, exhibiting medium to strong intensity. The presence of tridecane was confirmed in six different pollens.

The “sweet/baked/caramel/honey” flavor notes were attributable in general to oxygen‐containing heterocycles (tetramethyloxirane, furfural, furaneol), alcohols (2,6‐dimethylcyclohexanol, 2‐ethylhexanol) and aldehydes (*cis*‐2‐heptenal, *trans,trans*‐2,4‐hexadienal, *trans,trans*‐2,4‐dodecadienal).

Floral scent notes may be related mostly to the presence of terpenes (tetrahydrolinalool, linalool, geranyl acetone, *α*‐ionone, caryophyllene oxide, *β*‐ionone epoxide), but oxygen‐heterocycles (2‐furanmethanol), benzenoids (phenylethyl alcohol, p‐methylguaiacol) and esters (vinyl hexanoate, 2‐ethyl‐3‐hydroxyhexyl 2‐methylpropanoate, nonyl acetate, ethyl hexadecyl carbonate) have also carried this fragrance character in pollen samples. The floral fragrance of terpenes, with the exception of linalool, was observed to be of low intensity. In contrast, the flower‐like aroma of benzenoid compounds (phenylethyl alcohol, p‐methylguaiacol) was noted to range from weak to medium intensity.

The major group of aroma active volatiles was the terpenoids (32 compounds), which was mainly included monoterpenoids (20 constituents). Terpenes play an important role in forming pollen flavor, similar to other plant sources. It is also possible that some of the odorants may be derived from the bees themselves. The sunflower pollen sample contained the largest number of odor‐active terpenes: four components appeared with medium intensities possessing mushroom, herbal and honey notes. Additionally, four compounds were identified with weak intensities and green, herbal, and floral scents. The volatile fraction of rapeseed‐2 pollen was devoid of any volatile aroma‐active terpene compounds.

In the majority of the pollen samples, the highest peaks on the chromatograms were attributed to fatty acids (15.55%–50.58% of total peak area). These volatiles were characterized by green/sour, floral, fruity and spicy fragrance notes. Propanoic acid, 2‐methylpropanoic acid, pentanoic acid and hexanoic acid produced green and sourish odors. The predominant acids in bee pollens were acetic acid and hexanoic acid, which also exhibited sour and fruity odors in nearly all samples. The source of volatile fatty acids in pollen may be the pollenkitt (Dobson and Bergström 2000; Piskorski, Kroder, and Dorn 2011).

Thirteen odorous phenolic derivatives were perceived with diverse (fruity, herbal, sweet, floral) scent characters in bee pollens. The characteristic, pleasant flowery smell of phenylethyl alcohol and p‐methylguaiacol was recognizable in the volatile fractions of many pollen samples. Alkylbenzenes usually possess plastic‐like odor (Le Guen, Prost, and Demaimay 2000). Styrene as a potential contaminant has been identified in all samples and its plastic‐like smell was perceived in sweet cherry pollen with weak intensity.

Compounds containing heteroatom (sulfur or nitrogen) possessed herbal, green, sweet, baked, floral, fruity notes. The analysis of pollen samples revealed the presence of three sulfur constituents with aroma activity. The panelists reported an unfavorable and disagreeable olfactory impression associated with dimethyl sulfoxide in the case of traveler's joy pollen. The raw, green, herbal aroma of dipropyl disulfide has been identified in nine samples, with varying degrees of intensity. Additionally, dimethyl sulfide has been linked to green olfactory characteristics in three pollens. Certain nitrogen‐containing compounds were described as having sweet and herbal odor. Three aroma‐active nitrogen compounds were perceived in rapeseed and dandelion pollens, with weak intensities. One of the rapeseed pollens was found to contain methyl isocyanide, which exhibited a sweet odor character. It is unknown whether naturally occurring isocyanides exist in the plant kingdom; however, isothiocyanides are a common occurrence (Fahey, Zalcmann, and Talalay 2001; Pedras, Zheng, and Gadagi 2007). The biological function of these compounds is associated with the defensive mechanism of the organism that produces them (Kempf et al. 2010; Pedras and Yaya 2012; Višnjevec et al. 2021). Benzyl nitrile with weak rubber‐like smell was perceived in another rapeseed pollen. Nitriles can be volatile degradation products of glucosinolates in *Brassica* species (Hanschen et al. 2017). The analysis of dandelion pollen revealed the presence of 2‐pyrrolidinone, a compound with a faint herbal odor. The biochemical pathway of formation of this compound in the plant is currently unknown. However, several pyrrolizidine alkaloids are produced in the Asteraceae family, which includes *Taraxacum* species (Kempf et al. 2010; Kopp, Abdel‐Tawab, and Mizaikoff 2020). These have a large number of photolysis products with similar chemical structures (Yi et al. 2024).

Alcohols also played an indispensable role in the overall aroma quality of pollen, with 20 of the 36 compounds detected by GC–MS exhibiting aroma activity. The odor intensities of these constituents were predominantly low or medium. Alcohols, with the exception of those that are unsaturated or present in high concentrations, do not typically exert a significant influence on the flavor of foodstuffs. This is due to their high odor threshold.

Additionally, fatty acid degradation aldehydes, including pentanal, hexanal, octanal, and nonanal, possess aroma activity, exhibiting green, herbal, and fruity notes. These volatiles are a significant contributor to the aroma of pollen, as evidenced by the olfactory activity of 14 of the 21 identified aldehydes. Aldehydes typically impart a distinctive oxidized flavor profile (Le Guen, Prost, and Demaimay 2000).

The presence of ketones was identified as a contributing factor to the green, fruity and herbal notes observed in the pollen fragrance. Of the fifteen ketones detected by GC–MS, nine exhibited aroma activity. The most potent volatiles were identified as 3,5‐octadien‐2‐one (green, cucumber), 1‐penten‐3‐one (herbal), 1‐hydroxy‐2‐butanone (green, grass) and 2‐nonanone (herbal, green).

The esters were identified as the fragrance constituents with the strongest aroma activity, with 12 of the 27 identified odorous esters perceived with strong intensities in the samples. The esters imparted predominantly fruity (ethyl valerate, ethyl isovalerate, butyl acrylate, methyl hexanoate, ethyl hexanoate) and floral (vinyl hexanoate, 2‐ethyl‐3‐hydroxyhexyl 2‐methylpropanoate, nonyl acetate, ethyl hexadecyl carbonate) notes. Many of them appeared in the samples with medium to high intensity. The panelists have identified the presence of ethyl trichloroacetate in all samples, with the aroma being perceived as baked, roasted, and sweet with strong intensities. This compound was identified as the sole common aroma‐active constituent in the pollen samples. The precursor of this ester, trichloroacetic acid (TCA) is not known to occur in nature. However, its residues have been previously detected in the seeds of wheat, barley and oats following the application of a postemergence herbicide (Kadis et al. 1972), in fruits and vegetables irrigated with water containing trichloroacetic acid, and in field bean pods and seeds (Demint et al. 1975). Chloroacetic acids are widely used industrial chemicals (Reimann, Grob, and Frank 1996), which have been previously identified in the atmosphere and in precipitation (Frank et al. 1994; Clemens and Schöler 1992; Lorbeer, Hartl, and Kohlert 1994). This environmental pollutant may be formed by photooxidation of C_2_‐chlorocarbons as a secondary atmospheric pollutant (Franklin 1994), can be generated within plant cells by biooxidation of C_2_‐chlorocarbons (Weissflog et al. 2007) or in the soil by enzymatic chlorination of soil organic matter (Matucha et al. 2007). Owing to its widespread presence in the environment, TCA is often found in plant tissues (Cape et al. 2006; Forczek et al. 2008). The presence of the TCA derivative ethyl trichloroacetate in the volatile fraction of bee pollen is also likely to be a consequence of environmental contamination.

Despite the identification of a considerable number of hydrocarbons in pollen samples, only a limited number of these exhibited aroma activity, with only 10 volatiles displaying olfactory characteristics. The intensity of these VOCs was typically weak or medium, exhibiting sweet and fruity characteristics. However, tridecane, with a moderate to high intensity mushroom aroma, was identified in six pollen samples.

In addition to the aforementioned groups of compounds, some unknown aroma‐active odorants have also been perceived in bee pollen samples. These volatile constituents were not be detected by the mass spectrometer, but were present in quantities that could be discerned by the human olfactory system. The aroma profiles exhibited a remarkable diversity, encompassing a range of characteristics from sweet/caramel and fruity, floral to green, herbal. Bee pollens were not solely composed of aromatic compounds with a pleasant odor. Additionally, the judges identified several volatile organic compounds that exhibited a distinctly unpleasant odor. These compounds were 1‐[2‐(allyloxy)‐1‐methylethoxy]‐2‐propanol in rapeseed‐3 pollen, methyl isovalerate in rapeseed‐1 and dandelion pollens with strong intensities and isovaleric acid in rapeseed‐2, rapeseed‐3, sunflower and phacelia pollens. The aroma of bee pollens can therefore be attributed to the combined presence of favorable and unfavorable volatile compounds, which exhibit different odor characteristics and intensities.

#### 3.3.3 Key Odourants in the Bee Pollen Samples

Similarly to the findings of Dobson and Bergström (2000), an investigation into the aroma composition of bee pollen has revealed that each type of pollen possesses a distinctive odor profile. While numerous volatiles are common, there are also some flavor compounds that are exclusive to each pollen. The analysis revealed the presence of individual aroma constituents in all pollen samples (Table 4), which may serve as species‐specific markers for pollinators (Dobson, Groth, and Bergström 1996).

**TABLE 4:** Characteristic flavor compounds identified in bee pollens.

Pollen	Aroma compounds
Rapeseed‐1	Dimethyl disulfide, 2,4‐Dimethylpentanal, 6‐Methyl‐3,5‐heptadiene‐2‐one^a^, 2‐Pentenoic acid^a^
Rapeseed‐2	Benzyl nitrile^a^, 7‐Azabicyclo[4.2.2]deca‐2,4,9‐trien‐8‐one, 2‐Methylpentanol^a^, 6‐Methyl‐5‐hepten‐2‐ol, Dipropylene glycol, Ethyl 2‐ethoxy‐2‐hydroxyacetate^a^, Ethyl isovalerate^a^, 2‐Ethylbutanoic acid, Undecanoic acid
Rapeseed‐3	Isoborneol, Linalyl phenylacetate, Eugenol, 1‐[2‐(Allyloxy)‐1‐methylethoxy]‐2‐propanol^a^, *(E,Z)‐*3,6‐Nonadien‐1‐ol^a^, *(Z)‐*3‐Tetradecene, 1‐Tetradecen‐3‐yne, 1‐Chloro‐2‐methyl‐propane
Musk thistle	Caryophyllene oxide^a^, 5‐Methyl‐2‐furancarboxaldehyde^a^, 2‐Nonanone^a^, *γ*‐Valerolactone, 2‐Methyltetradecane^a^, 2,3‐Dimethyl‐2‐pentene^a^
Rock‐rose	Methyleugenol, 3‐Isopropyl‐4‐methyl‐1‐pentyn‐3‐ol, Acetopropyl acetate, Octyl acetate, Nonyl acetate^a^, 1‐Chlorohexadecane
Traveler's joy	Verbenone^a^, *p*‐Anisaldehyde, 2‐Cyclopentene‐1,4‐dione^a^, Tetradecane
Dropwort	(2‐Ethyloctyl)benzene, 4‐Methoxy‐1‐butanol^a^, Methyl 9‐oxononanoate^a^, Heptyl cyclobutanecarboxylate, 1‐Ethylpentyl acetate, 4‐Methylpentanoic acid, 1‐Pentadecene
Honey locust	*α*‐Copaene^a^, *γ*‐Muurolene, 2‐Butyl‐2‐octenal, 3,4,5,5‐Tetramethyl‐2‐cyclopenten‐1‐one, 2‐Ethylbutyl acetate^a^, Methoxypropyl acetate, 2,6,11‐Trimethyldodecane
Sunflower	*α*‐Terpinene, *β*‐Phellandrene, *β*‐Cymene, *α*‐Terpinolene, 4,8‐Dimethyl‐3,7‐nonadien‐2‐ol^a^, Farnesane, Tetrahydrolinalool^a^, *δ*‐Cadinol^a^, 4‐Terpineol^a^, 1‐Menthol^a^, *β*‐Chamigrene, Perilla aldehyde, *cis*‐Verbenol, Dihydrocarveol, 4,8‐Dimethyl‐3,7‐nonadien‐2‐ol, 3,7‐Dimethyl‐3‐octanol, 1‐Nonanol, 2‐Ethyl‐1‐hexanol^a^, 2‐Methylhexadecanol, Methyl‐10‐undecanoate, 3‐Hydroxy‐2,4,4‐trimethylpentyl isobutyrate^a^, *(E)‐*2‐Methyl‐2‐butenoic acid^a^
Red poppy	Ethyl iso‐allocholate, *β*‐Citral, *trans*‐2‐Pinanol, Geranyl acetate^a^, Citral, Ammonium oxalate, p‐Methylguaiacol^a^, 2‐Methyl‐2‐hexanol, Ethyl ketovalerate, Ethyl undecanoate, Ethyl hexadecyl carbonate^a^, 2‐Hydroxy‐*γ*‐butyrolactone
Phacelia	Isothujol^a^, Epoxy‐linalooloxide, 3‐Methyl‐1H‐pyrazole, 2‐Methylfuran, 2‐Ethylfuran, Tetramethyloxirane^a^, 2‐Heptanol, 2‐Octen‐1‐ol, 2‐Ethyl‐2‐hexen‐1‐ol^a^, Propanal^a^, Butanal, *(E)‐*2‐Hexenal^a^, *(Z)‐*2‐Heptenal^a^, *(E,E)‐*2,4‐Hexadienal^a^, *(E,E)‐*2,4‐Heptadienal, *(E,Z)‐*2,6‐Nonadienal^a^, *(E,E)‐*2,4‐Dodecadienal^a^, 1‐Penten‐3‐one^a^, *(E)‐*5‐Methyl‐4‐hepten‐3‐one, 1‐Hydroxy‐2‐butanone^a^, 2‐Methyl‐3‐octanone, Diethylacetic acid, *(E)‐*2‐Octene, 2‐Methyl‐2‐pentene, (1‐Methylethylidene)‐cyclohexane^a^, *(Z)‐*3‐Decen‐1‐yne, 3,5,5‐Trimethyl‐1‐hexene, 3‐Ethyl‐1,4‐hexadiene^a^, 1,9‐Dichlorononane
Sweet cherry	Dihydromyrcenol^a^, Lilac aldehyde A, Lilac aldehyde B, Lilac aldehyde C^a^, *β*‐Cyclocitral, Lilac alcohol A^a^, Lilac alcohol B, Lilac alcohol C, Lilac alcohol D^a^, 2,6‐Dimethylcyclohexanol^a^, 2,5‐Dimethyl‐3‐hexyne‐2,5‐diol, 2‐Undecenal, 2‐*t*‐Butyl‐6‐methyl‐5‐(3‐methylbutyl)[1,3]dioxan‐4‐one, Ethyl 8‐nonenoate^a^, Methyl 8‐nonenoate, Hexanoic acid, 5‐hydroxy‐3‐methyl‐, *δ*‐lactone, *γ*‐Vinyl‐*γ*‐valerolactone, 5‐Butylnonane
Wild blackberry	3‐Hexanone^a^
Dandelion	Borneol, Nerolidol^a^, 1‐Acetylpyrrolidine, Benzyl oleate, 3‐Methylpentanal, Methoxyacetic acid, 6‐Ethyl‐3‐octyl ester, Vinyl hexanoate^a^, Hexanoic acid, 2,2‐dimethylpropyl ester, Eicosane

^a^ Aroma‐active compounds detected by the olfactometric port.

A total of 123 VOCs were identified in the samples. Of these, 29 were found to be present in **phacelia**. Almost half of the aroma compounds were perceived by the panelists at the olfactometric port. The three **rapeseed** pollen samples were found to possess distinctive key odorants. It is noteable that none of these volatiles were identified in Chinese rapeseed pollen (Bi et al. 2024). In their examination of **poppy** flowers and pollen, Dobson, Groth, and Bergström (1996) observed a similarity in their aromas, noting a pungent smell to the human nose. This olfactory characteristic was found to derive mainly from the pollen. Of the identified unique fragrance components, terpenoid geranyl acetate was reported to have a slight deterrent effect on the landing frequency of pollinators (Dobson and Bergström 2000). In contrast to the findings of Dobson, Groth, and Bergström (1996), a considerable number of terpene compounds were identified in poppy pollen, exhibiting a notably intensity (19.08%), and the number of benzenoid compounds was also much higher. In certain species of Papaveraceae, the nitrogen‐containing floral scents may contribute to the olfactory properties that are specific to the species in question (Dahl, Wassgren, and Bergström 1990). The findings of Dobson, Groth, and Bergström (1996) indicated that the flowers and pollen of **dropwort** exhibited a pronounced olfactory stimulus when perceived by the human nose. In their study, only 10 aroma components were identified in pollen, with fatty acid derivatives and benzenoids representing the most prevalent. Furthermore, the sample was found to contain considerable quantities of the aforementioned aroma constituents. Terpenoids were also identified as significant fragrance constituents of dropwort pollen, as well, with a peak area ratio of nearly 10% and a dominant presence of limonene (6.39%). In the samples examined by Dobson, Groth, and Bergström (1996), these VOCs were not present in quantities sufficient to be detected. The unique fragrance constituents of **cherry** pollen included many linalool degradation products (lilac aldehydes and alcohols). These aroma compounds are important ingredients of the fragrance of many flowers and play a significant role in attracting pollinators (Dötterl et al. 2006). These odourans form from geranyl diphosphate via the enzyme linalool synthase, but many other enzymes (cytochrome P450, cyclase, reductase, hydrogenase) also contribute to their release (Chen et al. 2010).

The identification of the specific aroma compounds that exert a decisive influence on the olfactory characteristics of a given foodstuff is a challenging task, largely due to the vast array of compounds present in varying concentrations. The distinctive flavor of a food may be attributed to a relatively limited number of compounds present in trace amounts with a low odor threshold. The olfactory characteristics of pollen are the result of the combined presence of the fragrance constituents identified in them. As illustrated in Table 4, individual aroma components were identified in all pollen samples, with some also exhibiting aroma activity. However, no aroma characters were identified in the pollen during the olfactometric analysis that were identical to the perceptible scent of the pollen. As evidenced by the measurement data, the number of odor‐active compounds (26–42) was consistently lower than the number of detected aroma compounds (75–101). Nevertheless, the presence of all compounds in pollen is essential for the formation of the overall aroma. Furthermore, the interactions between the individual flavor components and the food matrix may also be a contributing factor in the development of the characteristic flavor.

The distinctive aroma of each pollen is shaped by volatile organic compounds that play a crucial role in defining its unique scent. These compounds are vital during the pollination process. To the best of our knowledge, the key odorants of the pollens mentioned in this study have not been previously reported in the literature as characteristic flavor constituents indicating botanical origin.

## 4 Conclusions

In order to investigate the volatile profile of different bee pollen samples, a headspace solid‐phase microextraction (HS‐SPME) coupled with gas chromatography–mass spectrometry (GC–MS) method was optimized and applied in this research work. In the initial stages of the process, the most effective SPME coating and the optimal sampling parameters (extraction time and temperature, desorption time) were selected. The pollens of different botanical origin exhibited considerable diversity in their aroma composition, with each pollen displaying a distinctive volatile profile. In this study, the number of aroma‐active constituents identified by GC‐O was considerably lower than that detected by GC–MS. The aroma activity observed in bee pollen is attributed to a volatile fraction representing 31.0%–48.3% of the total. In total, 75–101 odorants were identified in the 14 bee pollen samples of different botanical origin, which could be classified into 13 chemical classes. Of these, 26–42 were detected by the panelists. A total of 19 volatile compounds were identified in all samples of bee pollen. The VOCs did not display the typical pollen aroma; rather, the samples exhibited a combined olfactory effect resulting from the presence of multiple odorants. The most abundant VOCs in the majority of pollens were acids and esters with acetic acid, octanoic acid and hexanoic acid representing the major peaks on the chromatograms. The red poppy exhibited the greatest diversity of volatile constituents among the samples, while the honey locust displayed the lowest number of such constituents. In addition to the plant origin, it is possible that some of the aroma components (aldehydes with longer chain length, C9 <) in pollen may also derive from the bees' headspace. The most significant distinction between the bee pollens was the diverse ratio of the volatiles and chemical classes. Furthermore, the presence of specific odorants could be discerned, which may be associated with the botanical origin of the samples. A large number of terpenes were identified in pollens with a diverse range of scent characteristics, with 47.1% of them classified as odor‐active. The findings indicate that terpenoids play a pivotal role in shaping the overall aroma of bee pollen. The sensory attributes of this group of apiculture products have yet to be fully documented. Consequently, a study of the volatile profile could provide valuable additional insight. The present study is of significant importance in for determining the volatile profile of bee pollen, whether as a standalone product or when incorporated as an ingredient into other foodstuffs. Furthermore, our findings may contribute to the elucidation of the role of pollen volatiles in bees' preferences for different floral sources. Furthermore, this research represents a significant contribution to the field of food science, given the paucity of available data regarding the organoleptic properties of apiculture products beyond honey. A comprehensive examination of the volatile profile could therefore serve to enhance the existing body of knowledge in this area. Moreover, our findings demonstrate that the flavor wheel is an effective tool for providing a comprehensive sensory description of pollen pellets from individual plant species.

## Author Contributions

**Mariann Csóka:** conceptualization (equal), methodology (equal), visualization (equal), writing – original draft (equal), writing – review and editing (equal). **Rita Végh:** supervision (equal), visualization (equal), writing – original draft (equal), writing – review and editing (equal). **László Sipos:** funding acquisition (equal), supervision (equal), writing – review and editing (equal).

## Conflicts of Interest

The authors declare no conflicts of interest.

## Data Availability

The data that support the findings of this study are available from the corresponding author upon reasonable request.
